# Supplementary material for: Data-driven discovery of dimensionless numbers and governing laws from scarce measurements
Source: Nat Commun. 2022 Dec 8;13:7562. doi: 10.1038/s41467-022-35084-w (PMC9729234; doi:10.1038/s41467-022-35084-w)
Supplement: Supplementary file 1 — Supplementary Information [file 41467_2022_35084_MOESM1_ESM.pdf]

SUPPLEMENTARY INFORMATION: DATA-DRIVEN DISCOVERY OF DIMENSIONLESS NUMBERS AND GOVERNING LAWS FROM SCARCE MEASUREMENTS

Xiaoyu Xie
Department of Mechanical Engineering
Northwestern University
Evanston, IL 60208
xiaoyuxie2020@u.northwestern.edu

Arash Samaei
Department of Mechanical Engineering
Northwestern University
Evanston, IL 60208
arash.samaei@northwestern.edu

Jiachen Guo
Theoretical and Applied Mechanics
Northwestern University
Evanston, IL 60208
jiachenguo2020@u.northwestern.edu

Wing Kam Liu*
Department of Mechanical Engineering
Northwestern University
Evanston, IL 60208
w-liu@northwestern.edu

Zhengtao Gan*†
Department of Mechanical Engineering
Northwestern University
Evanston, IL 60208
zgan@utep.edu

December 4, 2022

ABSTRACT

Contents

1 Theory of dimensionless learning **2**

1.1 Problem statement 2

1.2 Construct dimensionless numbers 3

1.3 Determining the number of dimensionless numbers 4

1.4 Representation learning of scaling law 4

2 Summary of the demonstrated case studies **5**

3 Dimension matrix and basis vectors used in the main text **5**

4 Optimization schemes for dimensionless learning **7**

*Corresponding authors: zgan@utep.edu (Z. Gan), w-liu@northwestern.edu (W. Liu)
†Present address: Department of Aerospace and Mechanical Engineering, The University of Texas at El Paso, El Paso, TX 79968

4.1 Grid search-based two-level optimization 7

4.2 Gradient-based two-level optimization 8

4.3 Pattern search-based two-level optimization 8

4.4 Hyperparameter settings 9

4.5 Comparison of different optimization methods 14

5 Identified dimensionless numbers in the main text **14**

5.1 Vapor depression dynamics in laser-metal interaction. 14

5.2 Porosity formation in 3D printing of metals. 15

6 Robustness analysis of dimensionless learning **15**

6.1 Omit one necessary variable for independent parameters 17

6.2 Effects of adding extra variables to independent parameters 17

6.3 Comparison of different machine learning algorithms with dimensionless learning 18

6.3.1 Model generalization for different materials 20

6.3.2 Model generalization for different scales 20

7 Governing equation discovery from limited data **22**

7.1 Vorticity equation 22

7.1.1 Data generation and preprocessing 22

7.1.2 Symmetric invariant SINDy 23

7.1.3 Dimensionless learning 24

7.2 Navier-Stokes equation 25

7.3 Spring-mass-damper systems 27

7.3.1 Data generation and preprocessing 27

7.3.2 Temporary governing equation discovery 29

7.3.3 Dimensionless learning 29

7.4 Dynamic response of impulsively loaded plastic beams 30

7.4.1 Data generation and preprocessing 30

7.4.2 Temporary governing equation discovery 31

7.4.3 Dimensionless learning 31

7.5 Regression library suggestions 32

1 Theory of dimensionless learning

1.1 Problem statement

For a system with n independent variables and 1 dependent variable, the causal relationship can be written as:

$$p = f(p_1, p_2, ..., p_n). \tag{1}$$

These n independent variables can be described by r fundamental physical dimensions (e.g., length, mass, time, temperature).

We can construct a dimension matrix $\boldsymbol{D}$ for n independent variables:

$$\boldsymbol{D} = [\boldsymbol{v}(p_1), \boldsymbol{v}(p_2), .., \boldsymbol{v}(p_n)], \tag{2}$$

where $\boldsymbol{v}(p_i)$ is the dimension vector for the i-th independent variable with a length of r. The shape of dimension matrix $\boldsymbol{D}$ is $r \times n$. For example, if the independent variables of a system are related to four dimensions (length, mass, time, and temperature) and the i-th independent variable p_i is the velocity (its dimension is length per time), then the corresponding dimension vector $\boldsymbol{v}(p_i)$ is $[1, 0, -1, 0]^T$.

Based on classical dimensional analysis, Eqn. 1 can be rearranged in a dimensionless form:

$$\Pi = f(\Pi_1, \Pi_2, ..., \Pi_k). \tag{3}$$

According to Buckingham's Pi theorem [1], the number of dimensionless numbers k is equal to the number of variables n minus the number of dimensions r if the rank of the dimension matrix $\boldsymbol{D}$ is full. Although Buckingham's Pi theorem states a sufficient number of dimensionless numbers to describe a system, it does not state the necessary number of dimensionless numbers. Therefore, the proposed dimensionless learning aims to find m dominant dimensionless numbers from experimental data:

$$\Pi = f(\Pi_1, \Pi_2, ..., \Pi_m), \tag{4}$$

where m is less than or equal to k, $f(\cdot)$ is the best representation (i.e, scaling law) of the system, Π is the output dimensionless number, and $\{\Pi_1, \Pi_2, ..., \Pi_m\}$ are the input dimensionless numbers. Specifically, to find the j-th dimensionless number Π_j, we need to find a vector $\boldsymbol{w_j}$:

$$\Pi_j = p_1^{w_1^j} p_2^{w_2^j} ... p_n^{w_n^j}. \tag{5}$$

Therefore, finding m input dimensionless numbers is equivalent to finding $\{\boldsymbol{w_j}\}_{j=1}^m$. Dimensionless learning aims to find the $\{\boldsymbol{w_j}\}_{j=1}^m$ for input dimensionless numbers and the $\boldsymbol{w'}$ for output dimensionless number that satisfies two physical constraints. The first constraint is the principle of dimensional invariance:

$$\boldsymbol{D w_j} = \boldsymbol{0}. \tag{6}$$

Another constraint is that scaling law Eqn. 4 should have the best fit to a series of data points in the collected dataset.

1.2 Construct dimensionless numbers

The number of dimensions r is typically smaller than the number of independent variables n because there are only 7 fundamental dimensions [2] but a large number of variables of interest. Therefore, Eqn. 6 typically has infinite solutions $\boldsymbol{w_j}$ as the dimension matrix $\boldsymbol{D}$ is underdetermined [3].

To solve Eqn. 6, we can first find the K basis vectors $\boldsymbol{w_{bk}}$ of the solution $\boldsymbol{w}$:

$$\boldsymbol{D w_{bk}} = \boldsymbol{0}, k \in [1, K], \tag{7}$$

where $\{\boldsymbol{w_{b1}}, \boldsymbol{w_{b2}}, ..., \boldsymbol{w_{bK}}\}$ are linearly independent, and K is the number of basis vectors, which is equal to n minus rank($\boldsymbol{D}$).

To identify the basis vectors $\{\boldsymbol{w_{bi}}\}^K$ for $\boldsymbol{D w} = \boldsymbol{D} \sum_{\boldsymbol{i=1}}^{\boldsymbol{K}} \boldsymbol{\gamma_i w_{bi}} = \boldsymbol{0}$, we first need to rearrange the dimension matrix $\boldsymbol{D}$ in reduced row echelon form using Gaussian elimination. Then, we set each free variable to 1 and all others to 0 and substitute them to solve the system and obtain a basis vector. The number of free variables is equal to K.

We can represent $\boldsymbol{w^j}$ with a linear combination of basis vectors $\{\boldsymbol{w_{bk}}\}_{k=1}^K$. The Eqn. 6 can be written as:

$$\boldsymbol{D w_j} = \boldsymbol{D} \sum_i^K \gamma_i^j \boldsymbol{w_{bi}} = \boldsymbol{0}. \tag{8}$$

Once the basis coefficients $\boldsymbol{\gamma^j}$ have been found, the j-th dimensionless number Π_j for input variables can be obtained.

Similarly, the output dimensionless number Π for the dependent variable can be obtained. The only difference is that when constructing the output dimension matrix $\boldsymbol{D'}$, we must account for the dimension vector $\boldsymbol{v}(p)$ of the dependent variable p:

$$\boldsymbol{D'} = [\boldsymbol{v}(p), \boldsymbol{v}(p_1), \boldsymbol{v}(p_2), .., \boldsymbol{v}(p_n)]. \tag{9}$$

To find the output dimensionless number Π, we need to solve the following equation:

$$\boldsymbol{D'w'} = \boldsymbol{D'} \sum_{i}^{K'} \gamma'_i \boldsymbol{w'_{bi}} = \boldsymbol{0}, \tag{10}$$

where γ'_i is the i-th output basis coefficient, $\boldsymbol{w'_{bi}}$ is the i-th output basis vector, K' is the number of output basis vectors, and $\boldsymbol{w'}$ is a linear combination of $\{\boldsymbol{w'_{bi}}\}_{i=1}^{K'}$. Once obtaining the basis coefficients $\boldsymbol{\gamma'}$ for the output dependent variable p, we can have the corresponding output dimensionless number Π.

1.3 Determining the number of dimensionless numbers

Prior to training, we calculate all possible sparse output dimensionless numbers based on an L1 norm constraint for $\boldsymbol{w'}$, which is the sum of the absolute values for all elements in $\boldsymbol{w'}$. Typically, the L1 norm threshold for output dimensionless numbers is set to 5 (no more than 5 is acceptable). The motivation is that we assume the output dimensionless numbers should have a simple expression, despite the fact that the input dimensionless numbers can be relatively complex. The vapor depression dynamics problem in laser-metal interaction is a typical example. The output dimensionless number is a dimensionless length, keyhole aspect ratio, which is the keyhole depth to laser radius ratio. This output dimensionless number has an L1 norm of only 2, whereas the input dimensionless number (relatively more complex) has an L1 norm of 6.5.

Due to a low L1 norm threshold for the output, there are only a very few candidate output dimensionless numbers. For example, in the problem of keyhole dynamics (Section 2.2 of the main manuscript), there are seven candidate output dimensionless numbers, whereas in the case of turbulent Rayleigh-Bernard convection (Section 2.1 of the main manuscript), there are only two candidate output dimensionless numbers. Then we go through all of the possible output dimensionless numbers. Each time, we choose and fix one of the possible output dimensionless numbers. Next, we use two-level optimization methods to find the best input dimensionless numbers using grid search, gradient-based optimization, or pattern search-based optimization methods, as discussed in Section 4 of the Supplementary Information.

To determine the number of input dimensionless numbers, we perform the two-level optimization with one input dimensionless number and increase the number of input dimensionless numbers if the trained model underperforms, until we find the best representation, i.e., a model with a high R^2 score. This incremental learning scheme helps us to avoid unnecessary dimensionless numbers because if a small number of dimensionless numbers can represent the system, we will not increase the number of input dimensionless numbers. A flowchart of dimensionless learning with an incremental learning scheme is shown in Fig. 1. It is worth mentioning that, while cross-validation is not shown in Fig. 1, we recommend using it to tune the hyperparameters for the fitting function. For example, cross-validation can be used to determine the polynomial degree. There are also examples applying 5-fold cross-validation in Sections 3.4 and 5.3 of the SI.

1.4 Representation learning of scaling law

Another important task is to find the best representation $f(\cdot)$, also known as the scaling law. Depending on the problem, different fitting functions such as polynomial regression, neural network-based regression, symbolic regression, or tree-based regression methods can be used. In this section, we will demonstrate dimensionless learning with polynomial functions to simplify the demonstration.

When we use a polynomial with coefficients up to order 3, the causal relationship can be written as:

$$\Pi = f(\boldsymbol{\Pi}, \boldsymbol{\beta}) = \beta_0 + \sum_{i=1}^{m-1} \beta_i \Pi_i + \sum_{i=1}^{m-1} \sum_{j=1}^{m-1} \beta_{ij} \Pi_i \Pi_j + \sum_{i=1}^{m-1} \sum_{j=1}^{m-1} \sum_{k=1}^{m-1} \beta_{ijk} \Pi_i \Pi_j \Pi_k, \tag{11}$$

where $\boldsymbol{\Pi}$ is an input dimensionless number vector with a length of m based on independent variables, and $\boldsymbol{\beta}$ is polynomial coefficients.

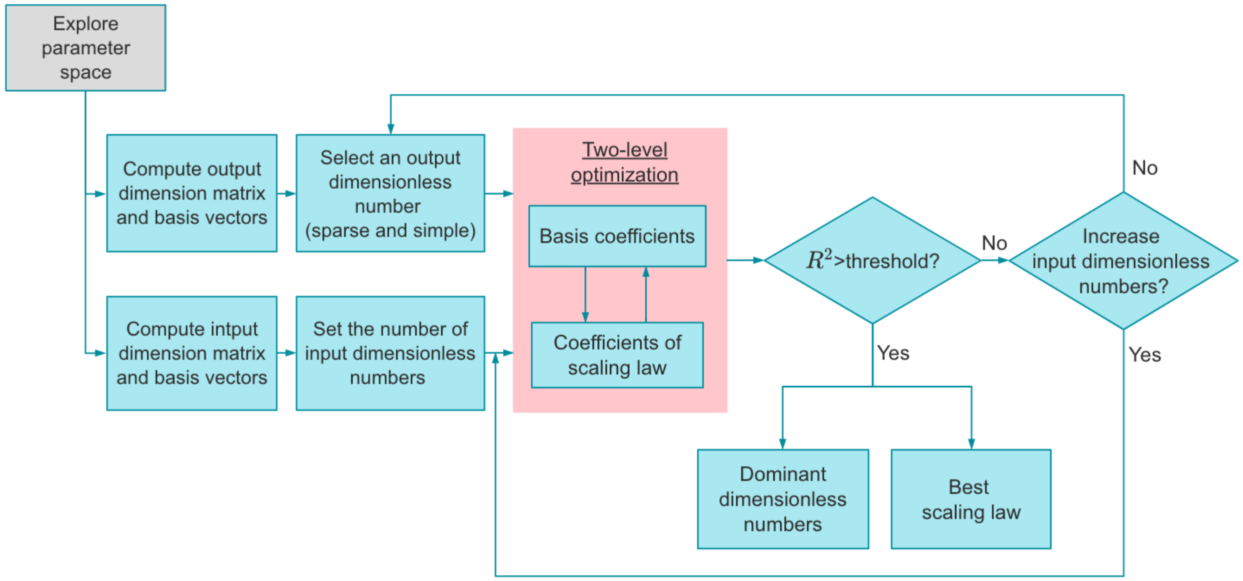

Supplementary Figure 1: **A flowchart for dimensionless learning**. After exploring parameter space for a complex system, the input and output dimension matrices and basis vectors are computed separately. By embedding dimensional invariance, we select a sparse output dimensionless number with a simple form (i.e., a small L1 norm) and random basis coefficients to initialize input dimensionless numbers. Then, using a two-level optimization method (grid search-based, gradient-based, or pattern search-based two-level optimization), we can obtain the optimal basis coefficients and scaling law given the chosen output dimensionless number. If the model performs well (R^2 exceeds the threshold), the dominant dimensionless numbers and the best scaling law can be determined. Otherwise, all forms of output dimensionless numbers must be explored. If the model continues to underperform, we increase the number of input dimensionless numbers and re-optimize the model. Otherwise, we switch to another possible output dimensionless number.

In summary, there are two sets of coefficients to be identified in order to automatically find m input dominant dimensionless numbers and one output dimensionless number:

1. Basis coefficients $\{\gamma^j\}_{j=1}^{m+1}$ for all dimensionless numbers;
2. Scaling law coefficients such as polynomial coefficients $\boldsymbol{\beta}$.

Following the flowchart in Fig. 1, a few possible dimensionless numbers and scaling laws can be obtained. Then, we select the optimal model based on the fitting performance (R^2 score) of the scaling law and the L1 norm of dimensionless numbers. We encourage a higher R^2 score and a lower L1 norm. In a few special situations, when different scaling laws have the same R^2 score and L1 norm, we choose the model with fewer variables. These criteria ensure that the scaling law is parsimonious and fits well on the dataset.

2 Summary of the demonstrated case studies

We summarize the details of the data and approaches to all the case studies in this paper in Supplementary Table 1 to avoid confusion for the readers.

3 Dimension matrix and basis vectors used in the main text

Vapor depression dynamics in laser-metal interaction. The dimension matrix $\boldsymbol{D}$ of this example is:

$$\begin{bmatrix} 2 & 1 & 1 & 2 & -3 & 2 & 0 \\ -3 & -1 & 0 & -1 & 0 & -2 & 0 \\ 1 & 0 & 0 & 0 & 1 & 0 & 0 \\ 0 & 0 & 0 & 0 & 0 & -1 & 1 \end{bmatrix} \tag{12}$$

Supplementary Table 1: **Summary of the demonstrated case studies using the proposed method in this paper.** It provides information regarding the data, noise level, and approaches to different problems demonstrated in different sections in the main manuscript and SI.

Type of knowledge discovery	Case study	Optimization method	Data Source	Noise in data	Identified Π	Identified model/equation	Sec. No.
Scaling law discovery	Turbulent Rayleigh-Bénard convection	Grid search; Gradient descent; Pattern search;	Experiments (literature)	Yes	Ra, Nu	Polynomial	Sec. 2.1
	Vapor depression dynamics	Grid search; Gradient descent; Pattern search;	Experiments (literature)	Yes	Ke, e*	Polynomial ($e^* = 0.12\mathrm{Ke} - 0.30$)	Sec. 2.2
	Porosity formation in 3D printing	Pattern search;	Experiments (literature)	Yes	$\mathrm{NED}\frac{d^3}{L^3}$, $\mathrm{NEP}\frac{L}{d}$	XGBoost (Tree-based model)	Sec. 2.3
Differential equation discovery	Vorticity equation	SINDy + dimensionless learning;	Simulation	1%	Re	$\frac{\partial\omega}{\partial t} = -u\frac{\partial\omega}{\partial x} - v\frac{\partial\omega}{\partial y} + \frac{1}{\mathrm{Re}}(\frac{\partial^2\omega}{\partial x^2} + \frac{\partial^2\omega}{\partial y^2})$	Sec. 2.4 & SI Sec. 7.1
	Navier-Stokes equation	SINDy + dimensionless learning;	Simulation	0.5%	Re, Eu	$\frac{\partial u^*}{\partial t} = -u\frac{\partial u^*}{\partial x} - v\frac{\partial u^*}{\partial y} - \mathrm{Eu}\frac{\partial p^*}{\partial x} + \frac{1}{\mathrm{Re}}(\frac{\partial^2 u^*}{\partial x^2} + \frac{\partial^2 u^*}{\partial y^2})$	SI Sec. 7.2
	Euler equation	SINDy + dimensionless learning;	Simulation	1%	Eu	$\frac{\partial u^*}{\partial t} = -u\frac{\partial u^*}{\partial x} - v\frac{\partial u^*}{\partial y} - \mathrm{Eu}\frac{\partial p^*}{\partial x}$	SI Sec. 7.2
	Spring-mass-damper system	SINDy + dimensionless learning;	Simulation	4%	N/A	$\frac{dx}{dt} = -\frac{k}{c}x - \frac{m}{c}\frac{d^2x}{dt^2}$	SI Sec. 7.3
	Dynamic loading beam systems	SINDy + dimensionless learning;	Simulation	2%	Rn	$\frac{\partial^2 M^*}{\partial x^{*2}} = \mathrm{Rn}\frac{\partial v^*}{\partial t^*}$	SI Sec. 7.4

The dimensions of the effective laser power ηP, the laser scan speed V_s, the laser beam radius r_0, the thermal diffusivity α, the material density ρ, the heat capacity C_p, and the difference between melting and ambient temperatures $T_l - T_0$ are represented from the first to the last column of the matrix, respectively. From the first to the last row, it represents the dimensions of length, time, mass, and temperature, respectively.

In this example, the dimension for the dependent variable, i.e., the keyhole depth e, is:

$$[1, 0, 0, 0]^T. \tag{13}$$

The computed input basis vectors $\boldsymbol{w_{b1}}$, $\boldsymbol{w_{b2}}$, and $\boldsymbol{w_{b3}}$ for this example are:

$$\boldsymbol{w_{b1}} = [0, 1, 1, -1, 0, 0, 0]^T, \tag{14}$$

$$\boldsymbol{w_{b2}} = [0, 2, 0, 0, 0, -1, -1]^T, \tag{15}$$

$$\boldsymbol{w_{b3}} = [1, -3, -2, 0, -1, 0, 0]^T. \tag{16}$$

Porosity formation in 3D printing of metals. The dimension matrix $\boldsymbol{D}$ for this example is:

$$\begin{bmatrix} 2 & 1 & 1 & -3 & 2 & 2 & 0 & 1 & 1 \\ -3 & -1 & 0 & 0 & -2 & -1 & 0 & 0 & 0 \\ 1 & 0 & 0 & 1 & 0 & 0 & 0 & 0 & 0 \\ 0 & 0 & 0 & 0 & -1 & 0 & 1 & 0 & 0 \end{bmatrix} \tag{17}$$

The columns of the matrix, from right to left, represent the dimensions of the effective laser power input $\eta_m P$, the laser scan speed V_s, the laser beam diameter d, the material density ρ, the material heat capacity C_p, the thermal diffusivity

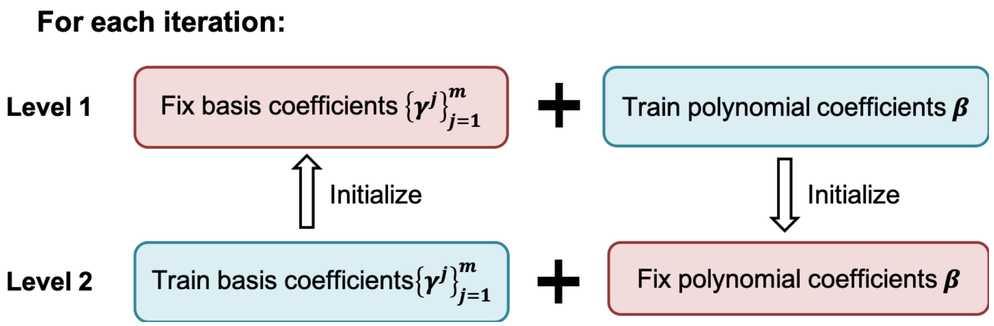

Supplementary Figure 2: **Two-level optimization method for dimensionless learning.** The blue blocks indicate that the coefficients are fixed, while the red blocks indicate that the coefficients are trainable.

α, the difference between melting and ambient temperatures $T_l - T_0$, the hatch spacing between the two adjacent laser scans H, and the layer thickness of the metallic powders L, respectively. The rows, from top to bottom, represent the dimensions of length, time, mass, and temperature, respectively.

In this example, the dimension of the dependent variable, which is the porosity volume fraction Φ, is:

$$[0,0,0,0]^T. \tag{18}$$

The computed input basis vectors $\boldsymbol{w_{b1}}$, $\boldsymbol{w_{b2}}$, $\boldsymbol{w_{b3}}$, $\boldsymbol{w_{b4}}$, and $\boldsymbol{w_{b5}}$ for this example are:

$$\boldsymbol{w_{b1}} = [0,2,0,0,-1,0,-1,0,0]^T, \tag{19}$$

$$\boldsymbol{w_{b2}} = [0,0,1,0,0,0,0,-1,0]^T, \tag{20}$$

$$\boldsymbol{w_{b3}} = [0,0,1,0,0,0,0,0,-1]^T, \tag{21}$$

$$\boldsymbol{w_{b4}} = [0,1,0,0,0.5,0,0.5,0,0]^T, \tag{22}$$

$$\boldsymbol{w_{b5}} = [1,-3,0,-1,0,0,0,0,-2]^T. \tag{23}$$

4 Optimization schemes for dimensionless learning

We developed three different optimization methods for dimensionless learning: grid search-based, gradient-based, and pattern search-based two-level optimization methods. The basic idea behind these two optimization methods is to optimize basis coefficients $\{\gamma^j\}_{j=1}^m$ and polynomial coefficients $\boldsymbol{\beta}$ sequentially at each iteration when the output dimensionless number is fixed, which is shown in Supplementary Figure 2. For each iteration, there are two levels of training procedures:

1. Level 1: train polynomial coefficients $\boldsymbol{\beta}$, while fix the basis coefficients $\{\gamma^j\}_{j=1}^m$;
2. Level 2: train the basis coefficients $\{\gamma^j\}_{j=1}^m$, while fix polynomial coefficients $\boldsymbol{\beta}$ as the newly obtained coefficients in level 1;

4.1 Grid search-based two-level optimization

For dimensionless learning, the key point is to identify the basis coefficients $\boldsymbol{\gamma}$ and polynomial coefficients $\boldsymbol{\beta}$. If a problem only involves a few basis coefficients (less than three) and a few input dimensionless numbers (less than two), grid search can work well to find the best coefficients, which is described in Algorithm 1. In this scenario, the grid interval can be relatively small such as 0.01, 0.02, or 0.04, etc. For example, there are three basis coefficients and one input dimensionless number for the turbulent Rayleigh-Bénard convection (Section 2.1 in the main manuscript) and the vapor depression dynamics in laser-metal interaction (Section 2.2 in the main manuscript). A fine grid interval of 0.04 is used in both cases. However, when there are multiple input dimensionless numbers or the number of basis coefficients is large, a large grid interval such as 0.5 is necessary to speed up the optimization. Also, the other two proposed two-level optimization methods in this paper are encouraged to be used to avoid the exponential growth of basis combinations.

Algorithm 1 Grid search-based two-level optimization for dimensionless learning

Require: Input variables $\boldsymbol{p}$, Dimension matrix $\boldsymbol{D}$, all possible sparse output dimensionless numbers $\{\Pi\}^H$, basis vectors $\{\boldsymbol{w_{bi}}\}^K$, the number of input dimensionless number M, the polynomial function degree Q, the grid interval d.

for $h \leftarrow 1$ to H **do** ▷ Loop for all possible output dimensionless numbers.
  for $i \leftarrow 1$ to M **do** ▷ Loop for all input dimensionless numbers.
    $\gamma_{i1} \leftarrow 1$.
    Divide [-2, 2] uniformly with a grid interval d for $\gamma_{i2}, \gamma_{i3}, ..., \gamma_{iK}$.
  end for
  Loop for all the basis vector combinations for M dimensionless numbers.
  For each combination, construct the input dimensionless numbers $\Pi_1, \Pi_2, ..., \Pi_M$.
  Fit $\Pi_h = f(\Pi_1, \Pi_2, ..., \Pi_M)$ with a polynomial with degree Q.
  Measure the fitting performance with R^2 score.
end for
Choose the best scaling law $\Pi_h = f(\Pi_1, \Pi_2, ..., \Pi_M)$ with a high R^2 score and a low L1 norm of dimensionless numbers.

4.2 Gradient-based two-level optimization

The gradient-based two-level optimization is shown in Algorithm 2.

Algorithm 2 Gradient-based two-level optimization for dimensionless learning

Require: Input variables $\boldsymbol{p}$, Dimension matrix $\boldsymbol{D}$, all possible sparse output dimensionless numbers $\{\Pi\}^H$, basis vectors $\{\boldsymbol{w_{bi}}\}^K$, the number of input dimensionless number M, the maximum number of iteration N, the polynomial function degree Q.

for $h \leftarrow 1$ to H **do** ▷ Loop for all possible output dimensionless numbers.
  for $i \leftarrow 1$ to M **do**
    $\gamma_{i1} \leftarrow 1$. ▷ Fix the i-th input dimensionless number's first coefficient.
    Initialize $\gamma_{i2}, \gamma_{i3}, ..., \gamma_{iK}$ with random numbers from [-2, 2]. ▷ Initial other basis vector coefficients.
  end for
  for $n \leftarrow 1$ to N **do**
    for $i \leftarrow 1$ to M **do** ▷ Obtain all temporary input dimensionless numbers.
      Obtain the temporary solution $\boldsymbol{w^i}$ of $\boldsymbol{Dw^i} = \boldsymbol{0}$, where $\boldsymbol{w^i} = \sum_{j=1}^{K} \gamma_{ij} \boldsymbol{w_{bj}}$
      Construct the i-th input dimensionless number Π_i^n=exp($\boldsymbol{w^{iT}}$log($\boldsymbol{p}$)) at the n-th iteration.
    end for
    Fit $\Pi_h = f(\Pi_1^n, \Pi_2^n, ..., \Pi_M^n)$ with a polynomial with degree Q. ▷ Level 1.
    Obtain the polynomial coefficients $\boldsymbol{\beta^n}$ at the n-th iteration.
    Fix the obtained $\boldsymbol{\beta^n}$, update $\{\gamma_{i1}^n, \gamma_{i2}^n, ..., \gamma_{iK}^n\}^M$ with BFGS algorithm [4]. ▷ Level 2.
  end for
end for

It is worth noting that if one dimensionless number in the input independent variables does not guarantee a high R^2 score, we will change the number of dimensionless numbers in the input independent variables to two and optimize again.

Supplementary Figures 3 and 4 show the gradient descent algorithm for the turbulent Rayleigh-Benard convection and keyhole dynamics cases, respectively. From these two figures, we notice that with enough iterations, most randomly initialized points can be optimized to the global optimum at 6000 iterations. The global optimal points in these two cases are the same, i.e., (1, 1). Therefore, we demonstrate that the gradient descent algorithm for two-level optimization is an efficient method for dimensionless learning. It is noted that these two figures only show points with a positive R^2 score in the last iteration.

4.3 Pattern search-based two-level optimization

A pattern search-based two-level optimization for a problem with two dimensionless numbers is shown in Algorithm 3.

Supplementary Figure 5 and 6 show the pattern search-based optimization results for the turbulent Rayleigh-Benard convection and keyhole dynamics problems, respectively. These figures contain three types of points: 1) red points

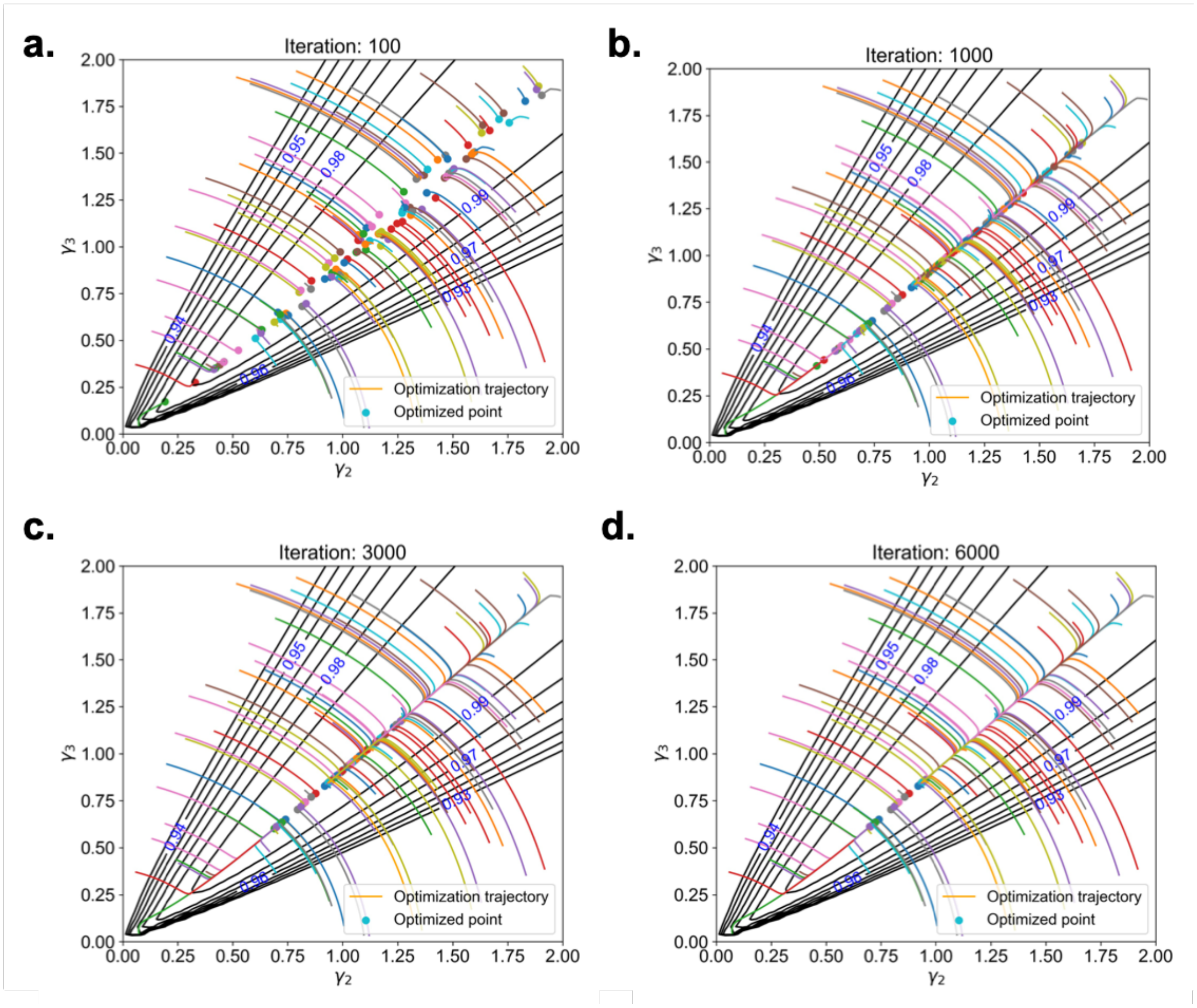

Supplementary Figure 3: **Gradient descent optimization for Turbulent Rayleigh-Benard convection.** Colored points indicate different initial basis coefficients. Colored curves indicate the corresponding optimization trajectories.

represent points that converge to the global optimal point (1, 1), 2) blue points represent points that end in locally optimal points, and 3) Green points represent points where the initial R^2 score is less than the threshold of 0.5.

Compared to gradient-based optimization in Supplementary Figures 3 and 4, pattern search-based optimization requires only 0.5% of the iterations that gradient descent optimization requires, and red initialized points can converge to the global optimal point (1, 1). By selecting the highest R^2 score, we can easily find the best solution, i.e., basis coefficients, from pattern search-based optimization. Therefore, pattern search-based optimization outperforms gradient-based optimization. One reason for its effectiveness is that we aim to find some simple dimensionless numbers, and a coarse grid for pattern search is adequate for locating the global optimal point.

4.4 Hyperparameter settings

The rationale behind selecting hyperparameters in this study can be summarized as follows: physical insights and experience, the tradeoff between accuracy and efficiency, and general trial and error selection or cross-validation.

First, based on physical insights and experience, dimensionless numbers should have simple forms. Thus, the exploration range of basis coefficients is set to [-2, 2], and the grid interval can be set to 0.01, 0.1, 0.25, or 0.5. These settings

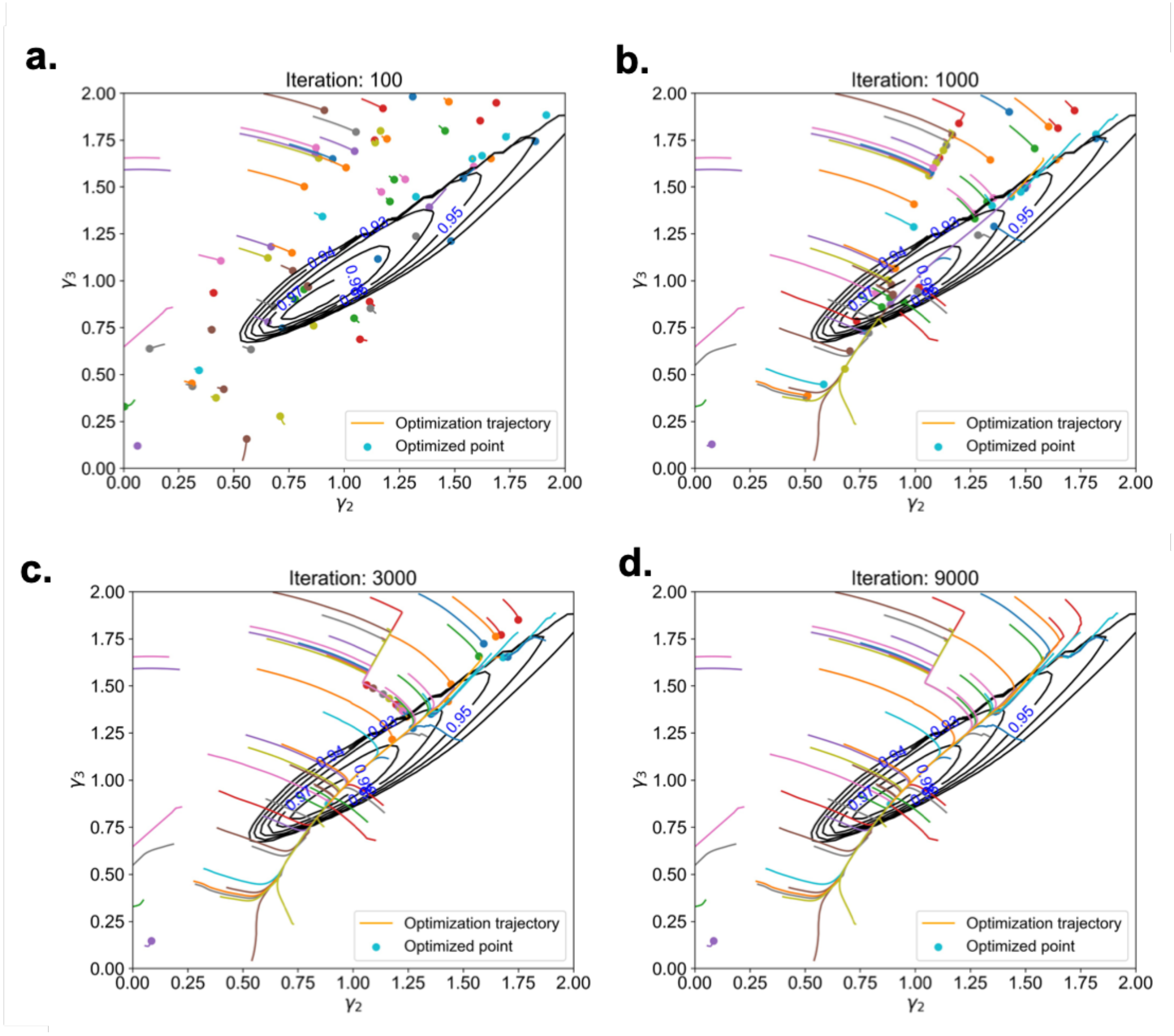

Supplementary Figure 4: **Gradient descent optimization for keyhole dynamics.** Colored points indicate different initial basis coefficients. Colored curves indicate the corresponding optimization trajectories. The grid range is [-2, 2], but only the range in [0, 2] is shown in the figure because this area has a higher R^2 score.

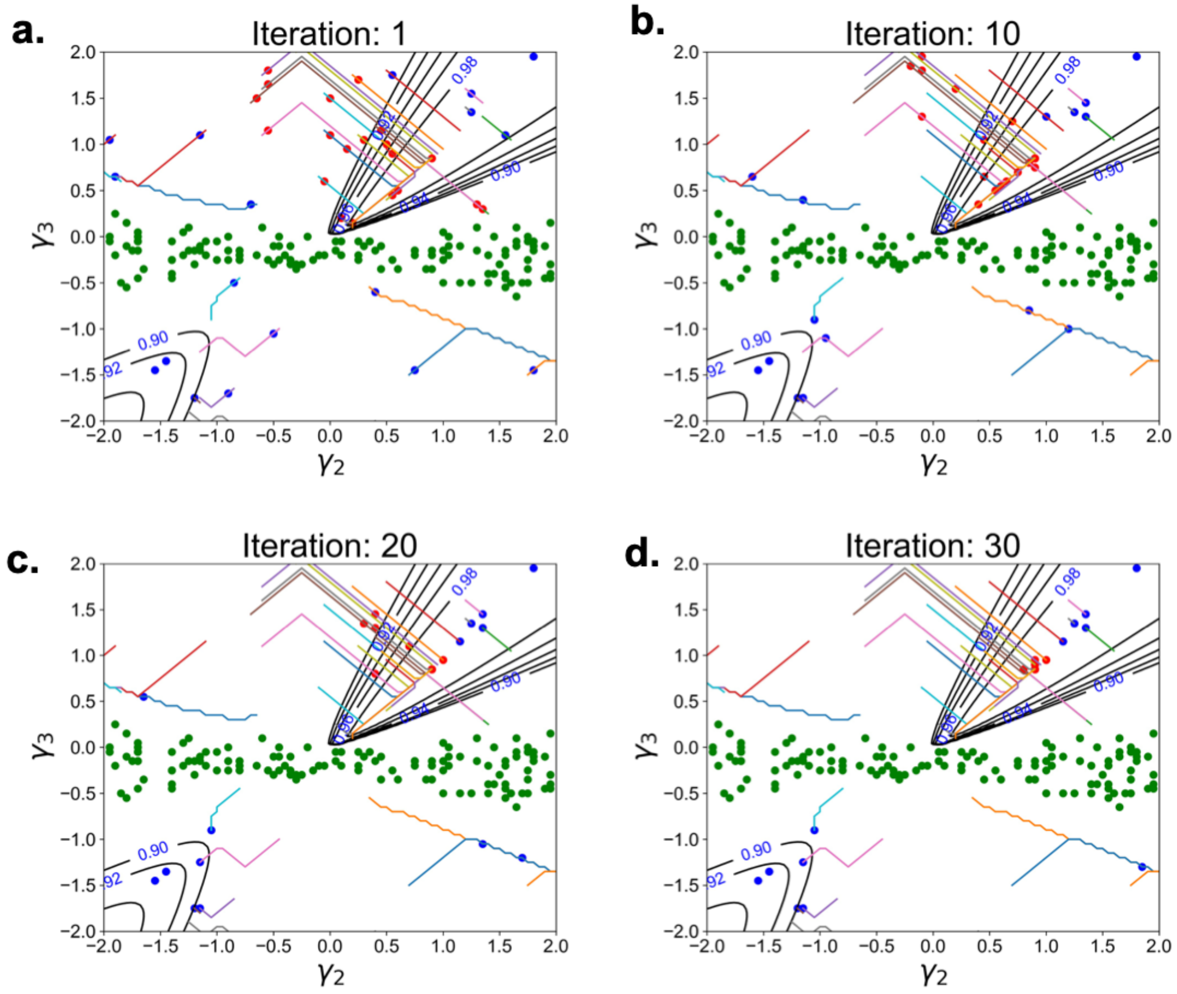

Supplementary Figure 5: **Pattern search optimization for turbulent Rayleigh-Benard convection.** Red points indicate the points that can be optimized to global optimal points. Blue points indicate the points that can only be optimized to local optimal points. Green points indicate the points that stayed at the initial position due to the low initial R^2 score. Colored curves indicate the optimization trajectories. Black contours indicate the range for different R^2 scores obtained from grid search two-level optimization. The grid range is [-2, 2] and the interval is 0.05.

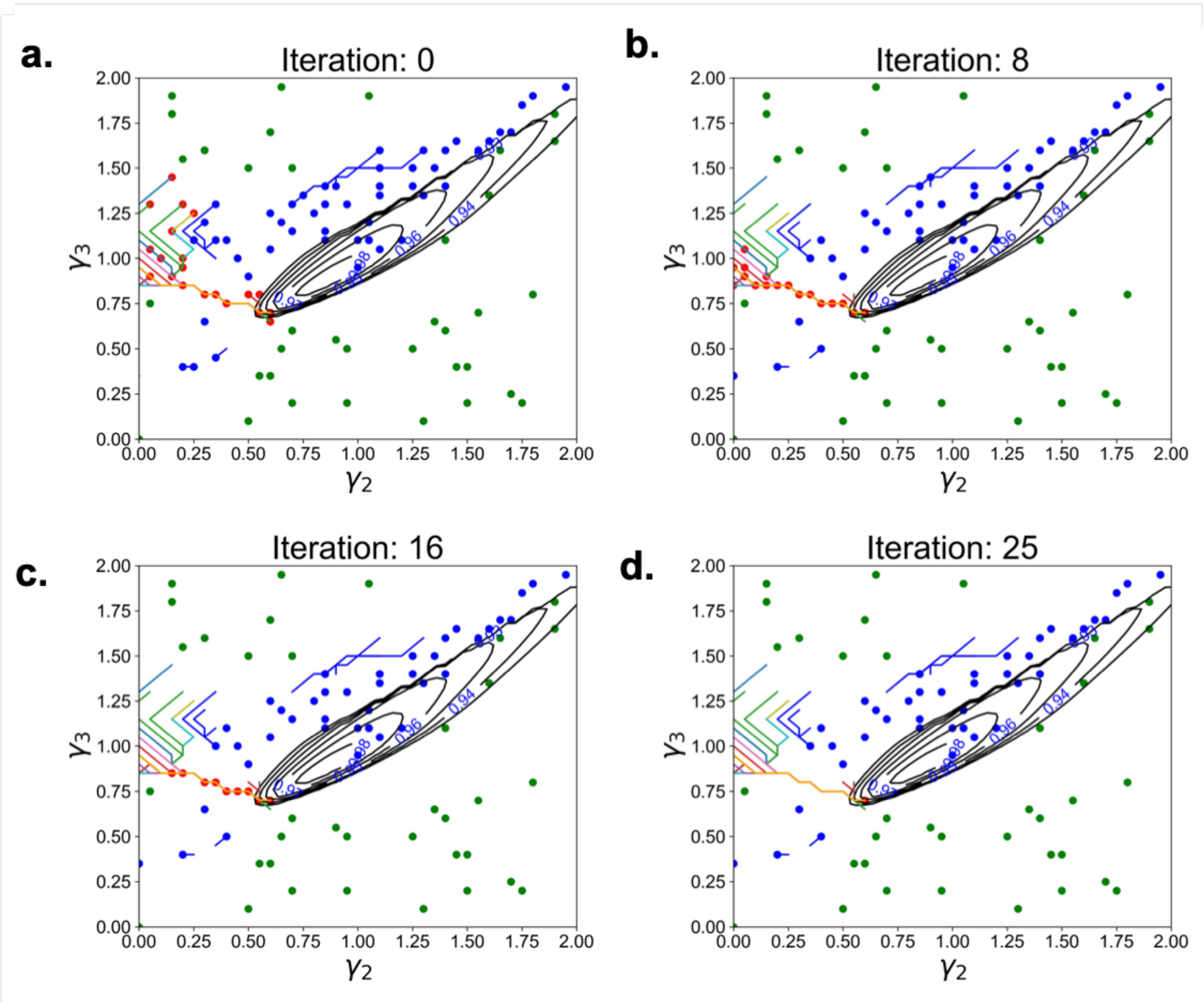

Supplementary Figure 6: **Pattern search optimization for keyhole dynamics.** Red points indicate points that can be optimized to global optimal points. Blue points are those that can only be optimized to local optimal points. Green points represent points that remained in the initial position due to the low initial R^2 score. The optimization trajectories are represented by colored curves. The black contours indicate the range of R^2 score obtained from grid search two-level optimization. The grid range is [-2, 2] and the interval is 0.05, but only the range in [0, 2] is shown in the figure because this area has a higher R^2 score.

Algorithm 3 Pattern search-based two-level optimization for dimensionless learning

Require: Input variables $\boldsymbol{p}$, Dimension matrix $\boldsymbol{D}$, all possible sparse output dimensionless numbers $\{\Pi\}^H$, basis vectors $\{\boldsymbol{w_{bi}}\}^K$, the number of input dimensionless number M, the maximum number of iteration N, polynomial function degree Q, a R^2 threshold for selection model r.

```
for h ← 1 to H do                              ▷ Loop for all possible output dimensionless numbers.
    for i ← 1 to M do
        γ_i1 ← 1.                               ▷ Fix the i-th input dimensionless number's first coefficient.
        Divide the domain for γ_i2, γ_i3, ..., γ_iK from [-2, 2] with an interval h = 0.05.
        Randomly initialize basis coefficients γ_i2, γ_i3, ..., γ_iK on the grid, which is called center point.
    end for
    for n ← 1 to N do
        for i ← 1 to M do                       ▷ Obtain all temporary input dimensionless numbers.
            Obtain the temporary solution w^i of Dw^i = 0: w^i = Σ_{j=1}^K γ_ij w_bj
            Construct the i-th input dimensionless number Π_i^n=exp(w^{iT} log(p))
        end for
        Use a polynomial with degree Q to fit the temporary representation based on the current input dimensionless
    numbers for the center point and calculate the corresponding R^2 score as R^2_center.
        if R^2_center < r then
            Continue.          ▷ Randomly choose another initial basis coefficients and calculate the new R^2_center.
        end if
        Calculate R^2 score for 3^K − 1 nearby points in a K dimensional Cartesian coordinate system.    ▷ Pattern
    search.
        Replace the current basis coefficients as the one with the highest R^2 score.
    end for
end for
```

help limit the power exponents of parameters in dimensionless numbers and avoid the identification of complex dimensionless numbers with many decimals.

Second, to achieve a balance between equation accuracy and algorithm efficiency, we recommend using a small grid interval (0.01 or 0.1) for problems with fewer basis coefficients (less than or equal to 4) and a large grid interval (0.25 or 0.5) for problems with more basis coefficients (more than 4). This is because small grid intervals can lead to inefficiency given a high dimensional solution space of basis coefficients. It is also worth mentioning that large grid intervals are usually sufficient to find the optimal dimensionless numbers because of the same assumption as in the first point: dimensionless numbers should be a power law with a simple form. In addition, to avoid being trapped by local minima in gradient-based and pattern search-based approaches, the number of initial points is set to 1,000 in this paper. However, because the computational cost of the proposed method is not high, more random initial points can be chosen.

Third, several hyperparameters should be determined through trial and error or cross-validation. We summarize them as below:

1. The L1 norm threshold for input dimensionless numbers can be different in problems. Higher L1 norm thresholds allow for the discovery of more complicated dimensionless numbers, and vice versa. Specifically, the thresholds in this study are 8, 9, and 13, respectively, for the problems in Sections 2.1 to 2.3 of the main manuscript.

2. In contrast to the input dimensionless numbers, which have a higher L1 norm threshold (greater than or equal to 8), the output dimensionless numbers in this paper are suggested to have a lower L1 norm, such as 5. This is because the output dimensionless numbers are usually related to the quantity of interest / dependent variable. We prefer a simple expression for the output dimensionless numbers so that the quantity of interest is easier to understand.

3. Cross-validation is an effective technique to determine hyperparameters. We demonstrate 5-fold cross-validation in determining the polynomial degree in the vapor depression dynamics problem in laser-material interaction, which is shown in Fig. 7. The R^2 score of a polynomial function with a degree of three is 0.9702, which is only slightly lower than that of a polynomial function with a degree of four (0.9708). To obtain a simple scaling law without degrading performance too much, we set the polynomial degree to three.

4. It is worth mentioning that because the BFGS optimization algorithm is used in the gradient-based optimization scheme, there is no need to manually select a learning rate or gradient descent step. The BFGS algorithm

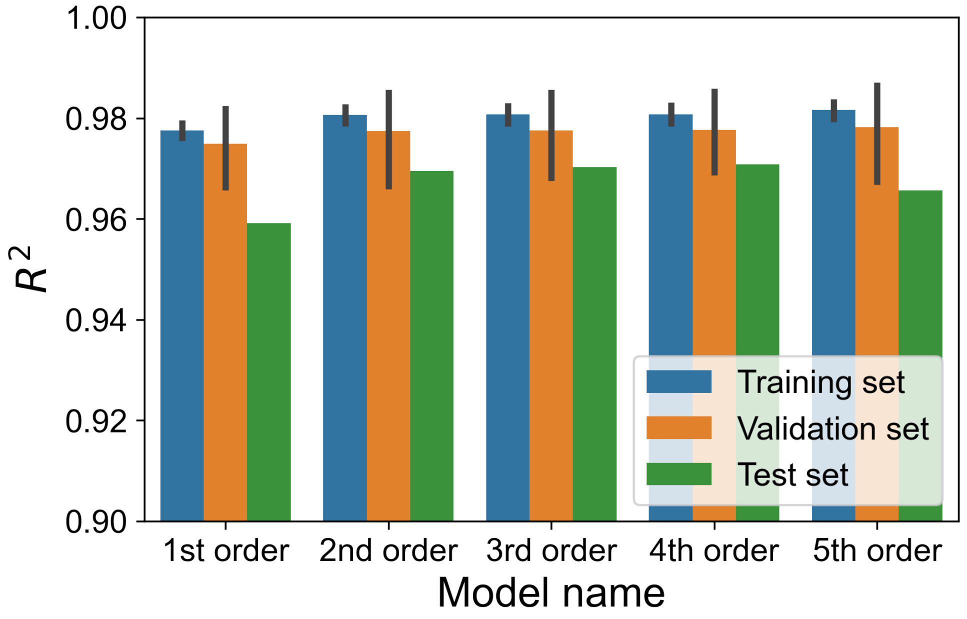

Supplementary Figure 7: **Polynomial degree identification for the keyhole dynamics problem using 5-fold cross-validation.** The error bar shows the 95% confidence interval.

is a second-order optimization algorithm that uses line search algorithms to determine the best learning rate for each iteration. If readers prefer to use other gradient descent methods, they need to select the appropriate learning rate and gradient step through trial and error.

There are two reasons to set the first basis coefficient γ_1 a constant value such as 0.5 or 1. First, a constant γ_1 helps to avoid the identification of equivalent dimensionless numbers. For example, assume that the best basis coefficients are $\{\gamma_i\}_{i=1}^K$ and that the corresponding dimensionless number is Π. Basis coefficients $\{C\gamma_i\}_{i=1}^K$ can also be used to formalize a dimensionless number Π^C, where C is a constant. By changing the fitting function, it could also achieve a high R^2 score. Therefore, we need to specify a constant for the first basis coefficient γ_1. Specifically, we can set γ_1 to 0.5 or 1 to achieve a small L1 norm of a dimensionless number. Other numbers to consider for γ_1 include -0.5 and -1. Second, by keeping one basis coefficient constant, all optimization methods can be made less computationally complex.

4.5 Comparison of different optimization methods

In this section, we summarize and highlight the differences between the three proposed optimization methods to clearly demonstrate their advantages and disadvantages. The detailed comparison is shown in Supplementary Table 2. It is worth noting that because we only want simple expressions of dimensionless numbers, zero-order optimization, such as the grid search-based approach or pattern search-based approach, is more suitable for finding the optimal basis coefficients, i.e., dimensionless numbers.

5 Identified dimensionless numbers in the main text

5.1 Vapor depression dynamics in laser-metal interaction.

As we mentioned in Section 1.3 of the Supplementary Information, we can obtain several output dimensionless numbers for a given problem. In keyhole dynamics, after going through all candidate output dimensionless numbers, we identify 7 sets of dimensionless numbers with a high R^2 score in both the training and test sets, which is shown in Supplementary Figure 8. The majority of their relationship is a straight line, with the exception of the last row, which is a quadratic curve. All of the dimensionless numbers performed greatly in both the training and test sets.

Furthermore, when we look at the best representation in the first row, i.e., $\mathrm{e}^* = 0.12\mathrm{Ke} - 0.3$, we notice that the bias is negligible when compared to the range of e^* ([0, 14]). After removing the bias, the R^2 score only slightly decreases

Supplementary Table 2: **Comparison of three proposed optimization methods.**

Optimization method	Advantages	Disadvantages
Grid search	a. Zero-order optimization. No need to compute gradients; b. Easy to implement; c. Applicable in low dimension problem; d. Flexible in the choice of grid interval;	a. The computational cost grows exponentially with increasing dimensionality; b. Need model selection after grid search;
Gradient descent	a. More capable of overcoming local minima; b. No need to manually select learning rate;	a. The optimized basis coefficients may not be integers or multiples of 0.5; b. Need more iterations to find the optimal solution;
Pattern search	a. Zero-order optimization. No need to compute gradients; b. Fewer iterations compared to gradient descent-based method; c. Flexible in the choice of step size (grid interval); d. Applicable for both low and high dimensional problems;	a. Easy to get stuck in local minima; b. Need more random initial points; c. Computationally expensive for high dimensional problems;

from 0.9826 to 0.9768. Therefore, the best representation can then be simplified as $\mathrm{e}^* = 0.12\mathrm{Ke}$. It is worth noting that all of the other dimensionless numbers (from the second to the last row in the Table of Supplementary Figure 8) can be regarded as some variants based on the $\mathrm{e}^* = 0.12\mathrm{Ke}$. This is due to the fact that a valid equation can still be obtained by multiplying a dimensionless ratio on both sides of $\mathrm{e}^* = 0.12\mathrm{Ke}$. For example, the input and output dimensionless numbers in the second row can be written as $\frac{r_0 V_s}{\alpha}\mathrm{Ke}$ and $\frac{r_0 V_s}{\alpha}\mathrm{e}^*$, respectively. The same coefficients $\frac{r_0 V_s}{\alpha}$ on the left and right sides of the equation can be canceled out.

5.2 Porosity formation in 3D printing of metals.

Since the dependent variable Φ is already dimensionless in this example, there is no other possible output dimensionless number. We only need to determine the best dimensionless numbers for the independent variables, i.e., input dimensionless numbers. We assume that there are two input dimensionless numbers. For each dimensionless number, there are five basis coefficients to be identified and we fix the first one for each dimensionless number. Therefore, in the first level of optimization, we need to determine eight basis coefficients in total.

Because determining the polynomial expression for two input dimensionless numbers is relatively difficult in this case, we use an XGBoost method [5] to capture the second-level relationships.

Here, we use a pattern search-based two-level optimization method. The identified dimensionless numbers are shown in Supplementary Table 3. The first two columns represent the two input dimensionless numbers. We notice that these four sets of dimensionless numbers are based on NED and $\mathrm{Ke_m} L_d^*$. By multiplying some simple dimensionless ratios for NED and $\mathrm{Ke_m} L_d^*$, the R^2 can be dramatically increased. For example, multiplying $\frac{d^3}{L^3}$ with NED and $\frac{L}{d}$ with $\mathrm{Ke_m} L_d^*$ (the third row of Supplementary Table 3) increases R^2 in the test set from 0.7581 to 0.9512.

6 Robustness analysis of dimensionless learning

The choice of parameter space has a significant impact on the discovered dominant dimensionless numbers and the best representation because changes in involved variables can directly change dimension matrix $\boldsymbol{D}$ and the corresponding solution $\boldsymbol{w}$. However, choosing an appropriate parameters space highly relies on researchers' experience and understanding of the problem. In this section, we analyze the robustness of dimensionless learning in the context

Π_1	Π	R^2 score (training set)	R^2 score (test set)
$\text{Ke} = \frac{\eta P}{(T_l - T_0)\rho C_p\sqrt{\alpha V_s r_0^3}}$	$e^* = \frac{e}{r_0}$	0.9763	0.9826
$\frac{r_0 V_s}{\alpha}\text{Ke} = \frac{\eta P\sqrt{V_s}}{(T_l - T_0)\rho C_p\sqrt{r_0\alpha^3}}$	$\frac{r_0 V_s}{\alpha}e^* = \frac{eV_s}{\alpha}$	0.9834	0.9852
$\frac{\eta P\sqrt{V_s^3}}{\sqrt{r_0\left(\alpha C_p(T_l - T_0)\right)^3\rho}}$	$\frac{eV_s^2}{\alpha\sqrt{C_p(T_l - T_0)}}$	0.9847	0.9845
$\frac{\eta P}{\sqrt{r_0 C_p V_s(T_l - T_0)\alpha^3}}$	$\frac{e\sqrt{C_p(T_l - T_0)}}{\alpha}$	0.9824	0.9936
$\frac{\eta P}{\sqrt{\alpha C_p(T_l - T_0)V_s^3 r_0^3\rho}}$	$\frac{e\sqrt{C_p(T_l - T_0)}}{V_s r_0}$	0.9806	0.9886
$\frac{\sqrt{(\eta P)^3}}{V_s C_p(T_l - T_0)\sqrt{r_0^3\alpha^3\rho^3}}$	$\frac{e^2\sqrt{C_p(T_l - T_0)}}{r_0\alpha}$	0.9642	0.9576
$\frac{\eta P}{r_0\alpha\rho C_p(T_l - T_0)}$	$\frac{e^2 V_s}{r_0\alpha}$	0.9809	0.9879

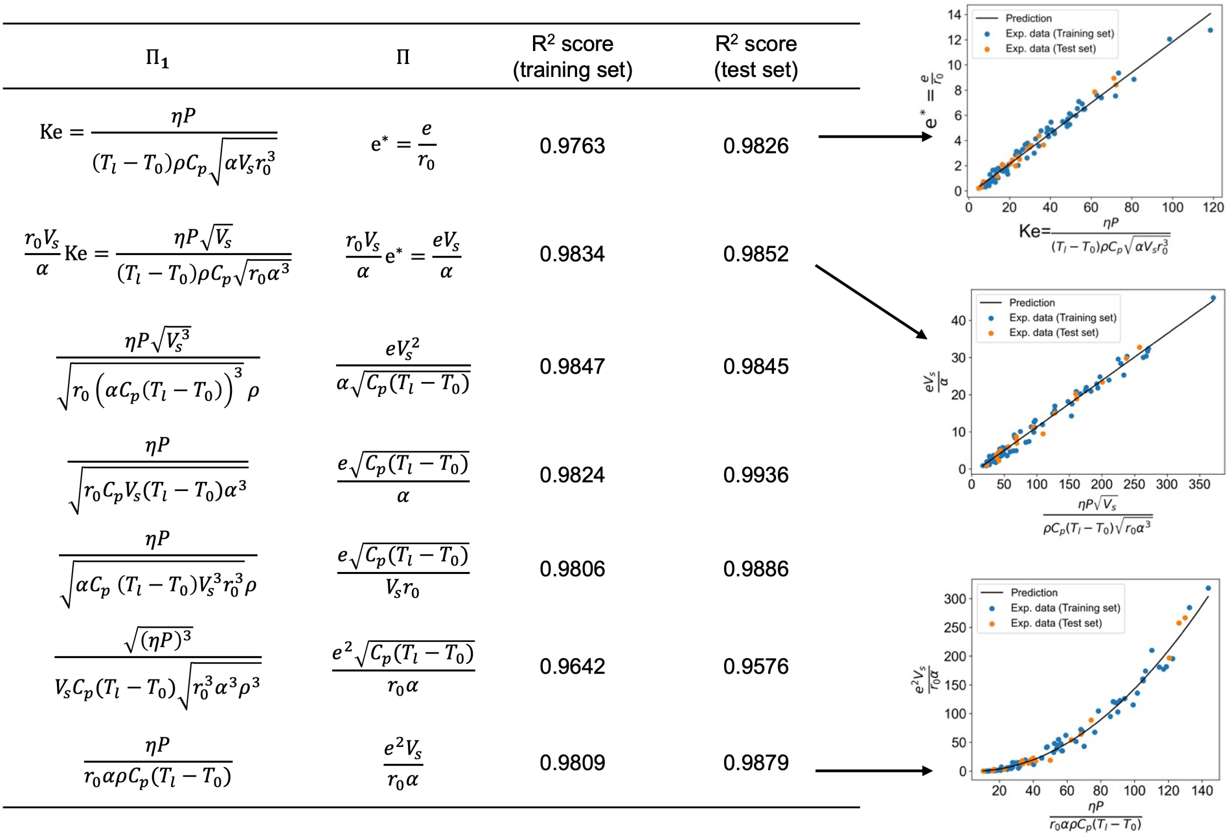

Supplementary Figure 8: **Identified dimensionless numbers for the keyhole dynamics problem.** The first and second columns contain the dimensionless numbers as input and output, respectively. The third and fourth columns are the corresponding model performances in the training and test sets, respectively.

Supplementary Table 3: **Identifying more dimensionless numbers for the porosity formation problem.** The first two columns are input dimensionless numbers, respectively. The third and fourth columns show the corresponding model performance in the training and test sets, respectively.

Π_1	Π_2	R^2 score (Training set)	R^2 score (Test set)
NED	NEP	0.9728	0.7581
NED	NEP $\frac{H}{d}$	0.9675	0.7025
NED $\frac{d^3}{L^3}$	NEP $\frac{L}{d}$	0.9561	0.9512
NED $\frac{d^2}{HL}$	NEP $\frac{HL\sqrt{C_p(T_l - T_0)}}{V_s d^2}$	0.9596	0.9380

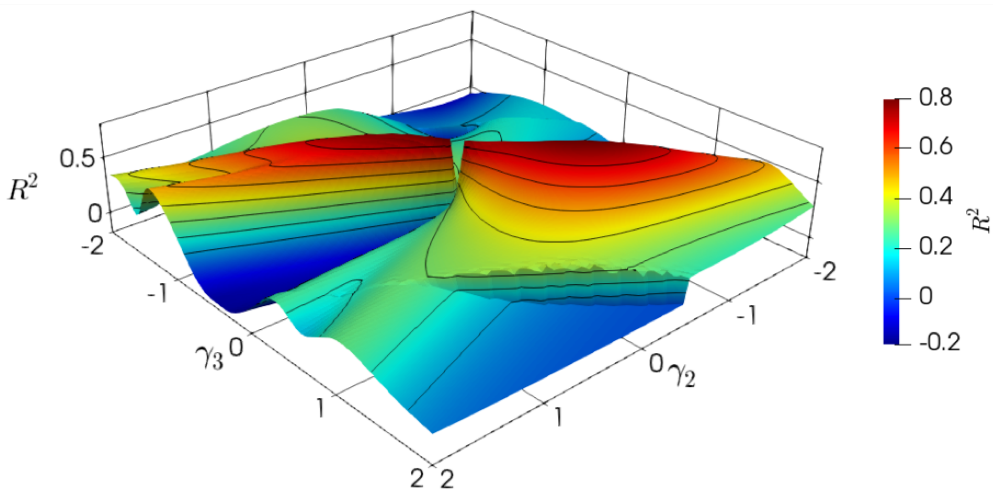

Supplementary Figure 9: R^2 **score for the test set for different combinations of basis vectors when we remove one necessary variable** α **from the input independent variables.**

of keyhole dynamics by omitting a necessary parameter or adding one additional parameter in the input independent variable. The results show that our proposed dimensionless learning method can not only find the best dimensionless number when additional variables are present but also guide us to include more variables when some important variables are missing.

6.1 Omit one necessary variable for independent parameters

Sometimes, due to a lack of knowledge or experience with a complex system, we may not take into account all necessary variables. In this section, we show how dimensionless learning can guide us to involve more variables in the analysis.

Here, we omit a necessary variable in the case of keyhole dynamics, namely the thermal diffusivity α, to see if the system can be expressed as a polynomial related to an input dimensionless number and keyhole aspect ratio e^*. The causal relationship can be written as:

$$\mathrm{e}^* = f(\eta P, V_s, r_0, \rho, C_p, T_l - T_0). \tag{24}$$

Since there are six independent variables and four fundamental dimensions, there are two basis vectors $\boldsymbol{w_{b1}}$ and $\boldsymbol{w_{b2}}$. Supplementary Figure 9 shows the R^2 score in the test set for different combinations of basis coefficients γ_1 and γ_2. The highest R^2 score is less than 0.80, indicating that these six independent variables are insufficient to construct a good scaling law to represent the system. Therefore, we know that we need to rethink the system to determine if we are missing any necessary variables or if there are no dominant dimensionless numbers in the system.

6.2 Effects of adding extra variables to independent parameters

First, we add the temperature difference between boiling and room temperatures $(T_v - T_0)$ as a new variable to the input independent variables, and then we fix the output dimensionless number as the keyhole aspect ratio e^*. The causal relationship is as follows:

$$\mathrm{e}^* = f(\eta P, V_s, r_0, \alpha, \rho, C_p, T_l - T_0, T_v - T_0). \tag{25}$$

Supplementary Table 4 shows the top five discovered dimensionless numbers with a high R^2 score. When the first two rows are compared, the second expression (keyhole number Ke) does not include the variable $(T_v - T_0)$. The R^2 score of keyhole number Ke is slightly lower in the training set, but it maintains the same R^2 score in the test set and has a simpler form. The dimensionless number expressions in the last three rows, on the other hand, become more complex after involving $(T_v - T_0)$ and perform poorly (lower R^2 score).

To quantitatively understand the effect of $(T_v - T_0)$ on the prediction of keyhole aspect ratio e*, we performed Sobol sensitivity analysis with an open-source python library called SALib [6, 7]. We begin by calculating the ranges for each

Supplementary Table 4: **Training and test set performances of different discovered dimensionless numbers for keyhole dynamics problem to analyze the effects of adding an extra variable $(T_v - T_0)$ into independent variables, where the output dimensionless numbers are fixed as the keyhole aspect ratio e^*.**

Π_1	R^2 score (Training set)	R^2 score (Test set)
$\frac{\eta P}{(T_v - T_0)^{0.25}(T_l - T_0)^{0.75}\rho C_p\sqrt{\alpha V_s r_0^3}}$	0.9863	0.9787
$\mathrm{Ke} = \frac{\eta P}{(T_l - T_0)\rho C_p\sqrt{\alpha V_s r_0^3}}$	0.9817	0.9787
$\frac{\eta P}{(T_v - T_0)^{0.125}(V_s(T_l - T_0))^{0.75}C_p^{0.875}\rho\sqrt{\alpha}}$	0.9737	0.9618
$\frac{\eta P(T_v - T_0)^{0.125}}{V_s^{0.75}C_p^{0.875}\rho(T_l - T_0)\sqrt{\alpha r_0^3}}$	0.9657	0.9564
$\frac{\eta P(T_v - T_0)^{1.125}}{V_s^{0.25}C_p^{0.875}\alpha r_0\rho(T_l - T_0)^{2.0}}$	0.9498	0.9510

input variable by examining the lower and upper bounds for the experimental data. Then, we generate 2^{10} model inputs by using the Sobol sequence [8], which is a quasi-random low-discrepancy sequence method for generating uniform samples. Next, we build a predictive model using the input dimensionless number $\frac{\eta P}{(T_v - T_0)^{0.25}(T_l - T_0)^{0.75}\rho C_p\sqrt{\alpha V_s r_0^3}}$ with the highest R2 scores in the training and test sets and evaluate the model predictions using the generated model inputs. Lastly, we use Sobol sensitivity analysis to analyze the model prediction and compute the sensitivity indices.

Fig. 10 shows the sensitivity analysis results for different possible variables. We can see that the sensitivity of $(T_v - T_0)$ is nearly zero, indicating that this variable has negligible influence on model prediction even though it is in the denominator of the input dimensionless number. Therefore, we choose keyhole number Ke as the input dimensionless number because it not only has a simpler form but also does not consider the unimportant variable. This also demonstrates that adding one additional variable $(T_v - T_0)$ has no effect on the dimensionless numbers discovered in this problem.

Second, we conduct another experiment in which we introduce a new variable, the latent heat of melting L_m, into the input independent variables and fix the output dimensionless number as the keyhole aspect ratio e^*. The system can be represented as:

$$\mathrm{e}^* = f(\eta P, V_s, r_0, \alpha, \rho, C_p, T_l - T_0, L_m). \tag{26}$$

Supplementary Table 5 compares the performance of the top five dimensionless numbers discovered with a high R^2 score. Even when an additional variable is taken into account, the keyhole number Ke remains the best dimensionless number because its expression is relatively simple and has a high R^2 score and a low MSE.

We also conduct sensitivity analysis for this experiment. In this case, we consider the input dimensionless number $\frac{\eta P}{L_m^{0.25}(C_p(T_l - T_0))^{0.75}\sqrt{\alpha V_s r_0^3}}$ in the third row of Supplementary Table 5. The model associated with this input dimensionless number has high R^2 scores in the training and test sets, albeit slightly lower than that of the model associated with keyhole number Ke.

Fig. 11 shows the sensitivity analysis results after including L_m in the dimensionless number expression. We notice that the sensitivity of L_m is very close to zero, indicating that adding this variable has no effect on the model prediction. Therefore, it is reasonable to omit this variable. In this experiment, we also demonstrate that the proposed dimensionless learning is robust even when an additional variable is considered.

6.3 Comparison of different machine learning algorithms with dimensionless learning

The out-of-bag error is a critical metric for machine learning algorithms, especially when dealing with problems with limited data. In this section, we compare the performance of five popular machine learning algorithms with our proposed

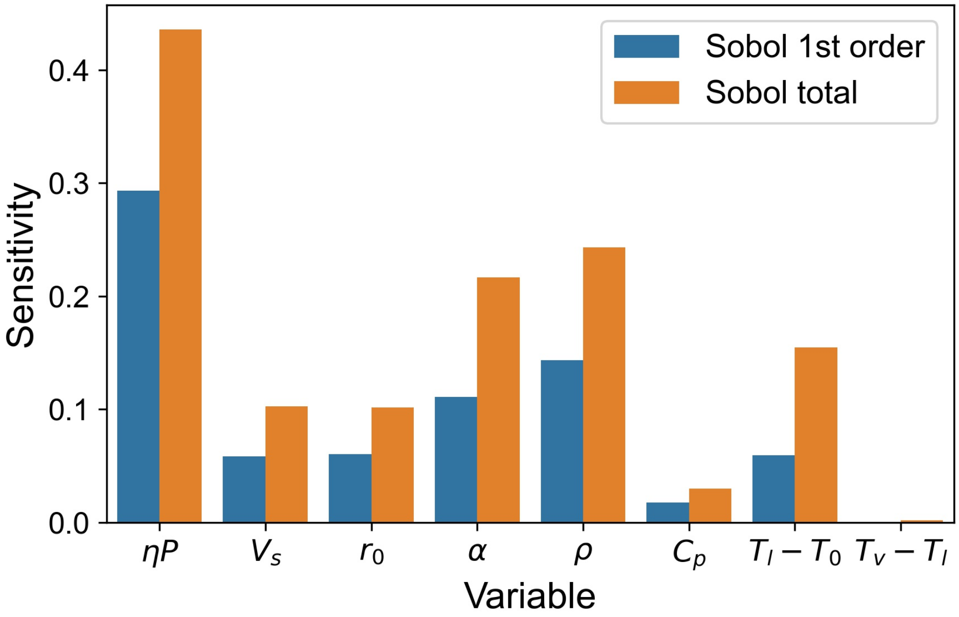

Supplementary Figure 10: **Sensitivity analysis for keyhole dynamics problem after involving an extra variable $T_v - T_0$.** Sobol 1st order indices measure how a single variable influences the output variable, while the Sobol total index measure how single and multiple input variables' affect the output variable. Sobol 1st order indices and total index means the variable has a stronger interaction between other variables.

Supplementary Table 5: **Performance of different discovered dimensionless numbers for the keyhole dynamics problem to perform sensitivity analysis for including an additional variable L_m into the independent variables, where the output dimensionless numbers are fixed as the keyhole aspect ratio e^*.**

Π_1	R^2 score (Training set)	R^2 score (Test set)
$\mathrm{Ke} = \frac{\eta P}{(T_l - T_0)\rho C_p \sqrt{\alpha V_s r_0^3}}$	0.9817	0.9787
$\frac{\eta P \sqrt{L_m}}{\alpha r_0 \rho (C_p(T_l - T_0))^{1.25} \sqrt{V_s}}$	0.8784	0.9729
$\frac{\eta P}{L_m^{0.25} \rho (C_p(T_l - T_0))^{0.75} \sqrt{\alpha V_s r_0^3}}$	0.9816	0.9701
$\frac{\eta P}{V_s^{0.75} (C_p(T_l - T_0))^{0.875} \rho \sqrt{\alpha r_0^3}}$	0.9707	0.9661
$\frac{\eta P L_m^{0.75}}{\alpha r_0 \rho (C_p(T_l - T_0))^{1.75}}$	0.8780	0.9490

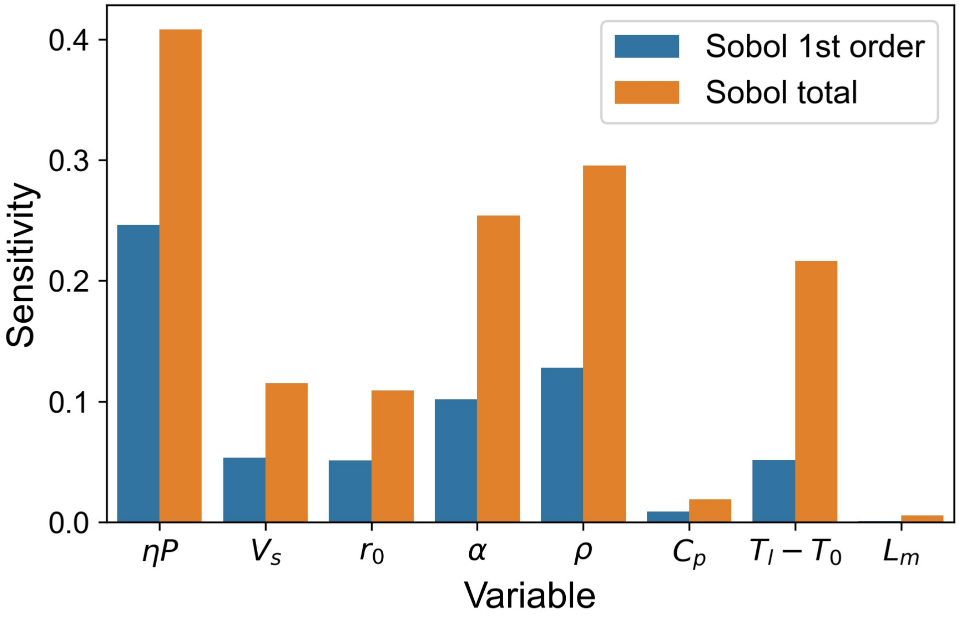

Supplementary Figure 11: **Sensitivity analysis for the keyhole dynamics problem with an additional variable L_m.**

method over two small datasets. The proposed method shows the best generalization in datasets with different materials and scales.

6.3.1 Model generalization for different materials

The first dataset is the same as the one used in Section 2.2 of the manuscript. This dataset contains three different materials: titanium alloy (Ti6Al4V), aluminum alloy (Al6061), and stainless steel (SS316). Here, we do not shuffle all the data and split it into training and test sets. We only use Ti6Al4V and Al6061 data in the training set, while other data (SS316) is used in the test set. 5-fold cross-validation is used to select the best parameters of each model.

Fig. 12 shows the R^2 score for each method. Even though the Feed Forward Neural Network (FFNN) achieves very good performance on the training and validation sets, the R^2 score for the test set is below -0.1. Random Forest (RF) has relatively high R^2 scores for training and validation, but it still performs poorly on the test set. On the other hand, the proposed method has the highest R^2 score on the test set, indicating that it has the best generalization in different materials. This improvement is the result of ensuring geometric, kinematic, and dynamic similarities based on similitude theory within different systems.

6.3.2 Model generalization for different scales

In this section, we use a rough pipe flow example to demonstrate that the proposed method has the best generalization ability in data with different scales. Pipe flow is a classic example to analyze fluid flow from laminar to turbulent [12]. The Reynolds number (Re) is widely used in fluid mechanics to distinguish between laminar and turbulent flow. In general, several measurements can be collected in the steady-state of a pipe flow system: fluid velocity v, dynamic viscosity μ, pipe diameter d, fluid density ρ, and pressure p.

Two types of synthetic data are created to build the dataset: small pipe flow data for the training and validation sets, and large pipe flow data for the test set. All the data are generated randomly. The detailed range of each parameter is shown in Supplementary Table 6.

Fig. 13 compares the performance of six regression models on the training, validation, and test sets. Because the test set consists entirely of large pipe flow rather than small pipe flow, all machine learning algorithms perform very poorly on the test set (below 0). On the other hand, the proposed method can perform very well on the test set. Even though this dataset is synthetic, it shows that the proposed method can be used for data with different scales.

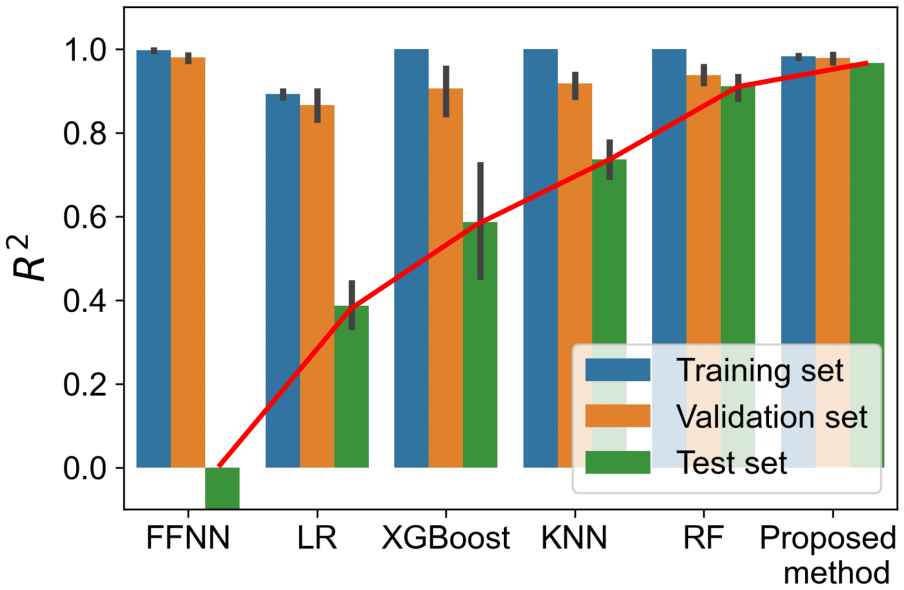

Supplementary Figure 12: **Comparisons of six candidate models to build a generalized model for the generalization of different materials.** The training and validation sets are associated with two different materials (i.e., titanium alloy (Ti6Al4V) and aluminum alloy (Al6061)), while the test set is for a third material (i.e., stainless steel (SS316)). The higher the R^2 score, the more accurate the model. The y-axis only shows R^2 scores greater than -0.1. The regression methods include Feed-forward Neural Network (FFNN) [9], Linear regression (LR), Extreme Gradient Boosting (XGBoost) [5], K-Nearest Neighbors (KNN) [10], Random Forest (RF) [11], and the proposed method. The error bar shows the 95% confidence interval.

Supplementary Table 6: **Different parameter ranges for small and large pipe flows.**

Source	Parameter	Range	Source	Parameter	Range
Small pipe	d	0.1 - 1 cm	Large pipe	d	10 - 100 cm
	μ	$1 \times 10^{-3} - 1 \times 10^{-2}$g/cm/s		μ	$1 \times 10^{-5} - 1 \times 10^{-3}$g/cm/s
	v	100 - 1×10^3 cm/s		v	1 - 10 cm/s
	ρ	$1 \times 10^{-3} - 1 \times 10^{-2}$g/cm^3		ρ	$1 \times 10^{-4} - 1 \times 10^{-3}$g/cm^3
	p	$1 \times 10^4 - 1 \times 10^5$Pa		p	$1 \times 10^4 - 1 \times 10^5$Pa

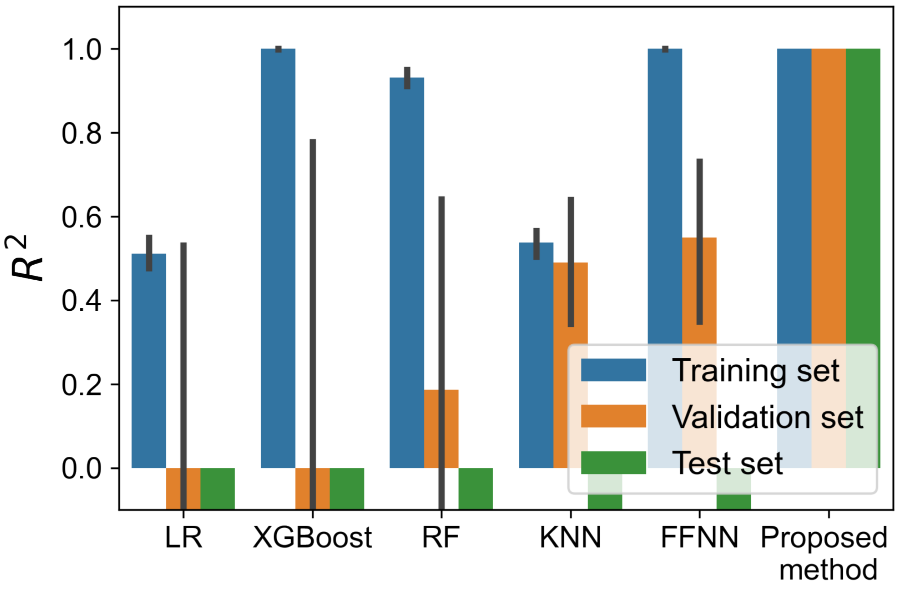

Supplementary Figure 13: **Comparisons of six candidate models to identify a generalized model for data from different scales.** The training and validation sets are generated using small pipe cases, while the test set is associated with large pipe cases. The higher the R^2 score, the more accurate the model is. The y-axis only shows $R2$ scores greater than -0.1. The error bar shows the 95% confidence interval.

7 Governing equation discovery from limited data

In this section, we integrate dimensionless learning with symmetric invariant SINDy (Section 7.1 and 7.2) or SINDy (Section 7.3 and 7.4) to identify governing equations as well as dimensionless numbers in fluid and solid mechanics problems. The identified Partial Differential Equations (PDEs) include the vorticity equation, Navier-Stokes equation, Euler equation, and the governing equation for spring-mass-damper systems and dynamic loading beam systems.

7.1 Vorticity equation

7.1.1 Data generation and preprocessing

The vorticity equation is a vorticity form of the Navier-Stokes equation that describes fluid flow by using the evolution of vorticity. To generate the original data, we conducted three sets of simulations for the Kármán vortex street problem with three circular cylinders using Flow3D [13]. The geometry parameters include the diameter of the cylinders l, the domain length in x direction l_x, and the domain length in y direction l_y. The operational and material parameters considered for this problem include the inlet flow velocity V, the fluid dynamic viscosity μ, the fluid density ρ, and the pressure difference between upstream and downstream p_0. Other simulation parameters include the number of mesh cells in x and y directions (N_x and N_y). The detailed information on these parameters is shown in Supplementary Table 7.

To identify the governing equation from data, 100 time-step snapshots are prepared and sparsely sampled in time and space domains. Fig. 14 shows a typical snapshot and the sampling region. In the sampling region, 4,000 data points are randomly sampled. Each data point includes values for six main variables in order to form the dataset: a specific time step (t), two spatial Cartesian coordinates (x and y), and three time-varying measurements (velocity in x direction u, velocity in y direction v, and vorticity ω). These six variables are non-dimensionalized in order to form dimensionless homogeneous differential equations by the following characteristic variables: characteristic length l (the cylinder diameter), characteristic velocity V (inlet flow velocity), characteristic time $t_{ref} = V/l$, and characteristic vorticity $\omega_{ref} = l/V$.

Supplementary Table 7: **Simulation parameters of fluid flow through three circular cylinders to identify the vorticity equation.**

Parameter<br>Unit	l<br>cm	V<br>cm/s	μ<br>kg/cm/s	ρ<br>g/cm^3	p_0<br>Pa	Re<br>1	l_x<br>cm	l_y<br>cm	N_x<br>1	N_y<br>1
Case 1	0.0850	68.00	0.1156	0.0010	1.30×10^4	50	0.9	0.4	500	222
Case 2	0.1000	90.00	0.1100	0.0011	8.42×10^4	90	0.9	0.4	500	222
Case 3	0.1000	100.00	0.1100	0.0011	8.53×10^4	100	0.9	0.4	500	222

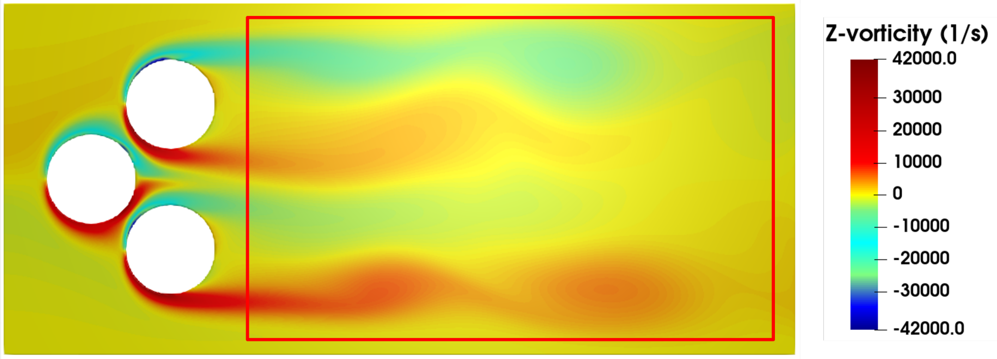

Supplementary Figure 14: **Vorticity contour plot from a simulation with Re=100.** The red color rectangle represents the sampling region.

7.1.2 Symmetric invariant SINDy

Fig. 15 shows a schematic of concatenating the sparse regression terms from the original and transformed data. Symmetric invariant SINDy relies on this operation to implicitly incorporate symmetric invariance into the algorithm. The detailed procedures are described below. First, we flip the original data from each simulation along $y = x$ to obtain symmetrically transformed data, allowing to augment training data. Note that this symmetric transformation fundamentally maintains system properties invariant as it applies to the entire system. Second, the original and transformed data are written for this dynamic system using sparse regression and then concatenated to further determine the sparse vector of coefficients. For example, the columns of $\frac{\partial \omega}{\partial t}$ and $\frac{\partial \omega}{\partial x}$ in Fig. 15 are concatenated with $\frac{\partial \omega'}{\partial t}$ and $\frac{\partial \omega'}{\partial x'}$, respectively. The entire concatenated data are used in the optimization, which means the algorithm is optimized with a single "batch" that contains all the training data rather than a mini-batch that contains only a portion of the training data.

There are three implicit benefits for sparse regression because of this concatenation operation. First, terms with the same absolute values in the original and transformed data must have the same coefficients. For example, the coefficients for $\frac{\partial \omega}{\partial x}$ (the fourth column) and $\frac{\partial \omega}{\partial y}$ (the fifth column) must be the same ($\xi_5 = \xi_6$). Second, the learnable coefficients are reduced because of the first benefit, which reduces the solution space and the required number of training data accordingly. Third, the augmented data as a result of this concatenation enables the method to avoid overfitting, because data augmentation is one of the key regularization techniques to prevent overfitting [14]. With this concatenation operation, the training data is doubled, which improves the learning accuracy for the regression coefficients.

The library of candidate terms includes the following 29 standard terms for a second-order partial differential equation:

$$\begin{aligned}\frac{\partial \omega}{\partial t} = [u, v, \omega, \frac{\partial \omega}{\partial x}, \frac{\partial \omega}{\partial y}, \frac{\partial^2 \omega}{\partial x^2}, \frac{\partial^2 \omega}{\partial y^2}, \frac{\partial^2 \omega}{\partial x \partial y}, uu, uv, u\omega, u\frac{\partial \omega}{\partial x}, u\frac{\partial \omega}{\partial y}, u\frac{\partial^2 \omega}{\partial x^2}, u\frac{\partial^2 \omega}{\partial y^2}, u\frac{\partial^2 \omega}{\partial x \partial y}, \\ vv, v\omega, v\frac{\partial \omega}{\partial x}, v\frac{\partial \omega}{\partial y}, v\frac{\partial^2 \omega}{\partial x^2}, v\frac{\partial^2 \omega}{\partial y^2}, v\frac{\partial^2 \omega}{\partial x \partial y}, \omega\omega, \omega\frac{\partial \omega}{\partial x}, \omega\frac{\partial \omega}{\partial y}, \omega\frac{\partial^2 \omega}{\partial x^2}, \omega\frac{\partial^2 \omega}{\partial y^2}, \omega\frac{\partial^2 \omega}{\partial x \partial y},]\boldsymbol{\xi},\end{aligned} \tag{27}$$

where the dimension of $\boldsymbol{\xi}$ is 29×1 and the derivative terms are calculated by a local polynomial interpolation approach described in [15, 16]. A second-order polynomial is fitted to each data point using eight neighboring points, and the derivatives can be calculated by the fitted polynomial.

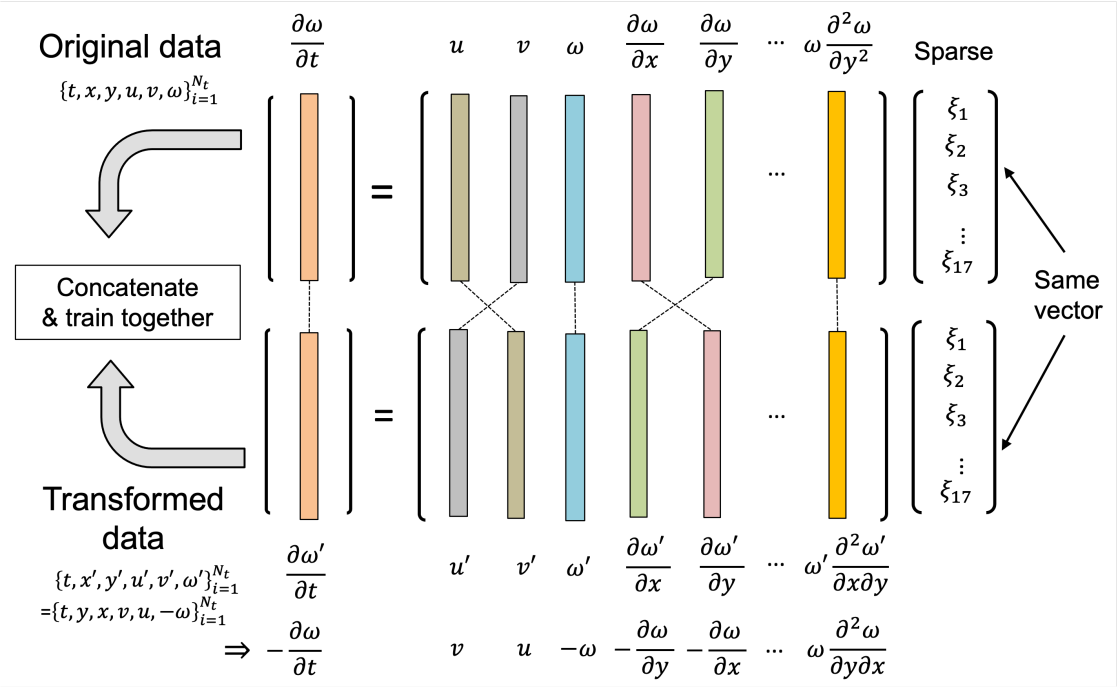

Supplementary Figure 15: **Concatenating the regression library for symmetric invariant SINDy from the original and transformed data.** Columns with the same color have the same absolute value.

Supplementary Table 8: **Dataset including computed non-zero regression coefficients and parameters for the vorticity simulation.**

Parameter<br>Unit	l<br>cm	V<br>cm/s	μ<br>kg/cm/s	ρ<br>g/cm^3	p_0<br>Pa	Re<br>1	$\xi_{12} = \xi_{19}$<br>1	$\xi_6 = \xi_7$<br>1
Case 1	0.0850	68.00	0.1156	0.0010	1.30×10^3	50	-0.9925	0.0212
Case 2	0.1000	90.00	0.1100	0.0011	8.42×10^4	90	-0.9909	0.0126
Case 3	0.1000	100.00	0.1100	0.0011	8.53×10^4	100	-0.9941	0.0110

To find sparse regression coefficients $\boldsymbol{\xi}$, we use a sequential threshold least-squares algorithm [15]. After optimization, each case will have only four non-zero regression coefficients. Thus, we are able to construct a non-zero coefficient dataset in combination with the parameters (and their values) listed in Supplementary Table 7 for the three different cases, as shown in Supplementary Table 8.

From Supplementary Table 8, we notice that $\xi_{12} = \xi_{19}$ is very close to a constant value (-1), while $\xi_6 = \xi_7$ varies with different cases. Therefore, the temporary governing equation can be simplified as:

$$\frac{\partial \omega}{\partial t} = -u\frac{\partial \omega}{\partial x} - v\frac{\partial \omega}{\partial y} + \xi_6(\frac{\partial^2 \omega}{\partial x^2} + \frac{\partial^2 \omega}{\partial y^2}) \tag{28}$$

7.1.3 Dimensionless learning

To obtain a consistent governing equation, we apply dimensionless learning to identify the expression of ξ_6 and ξ_7. The causal relationship can be expressed as:

$$\xi_6 = \xi_7 = f(\mu, l, V, \rho, p_0). \tag{29}$$

The dimension matrix $\boldsymbol{D}$, as described in Section 1 of the SI, for Eqns. 29 is obtained as follows:

$$\begin{bmatrix} -1 & 1 & 1 & -3 & -1 \\ -1 & 0 & -1 & 0 & -2 \\ 1 & 0 & 0 & 1 & 1 \end{bmatrix} \tag{30}$$

The columns of the matrix, from right to left, represent the dimensions of μ, l, V, ρ, and p_0 respectively. The rows, from top to bottom, represent the dimensions of length, time, and mass, respectively.

Unlike Sections 2.1 and 2.2 of the main manuscript, where a polynomial is used to fit the relationship between dimensionless numbers, we can simplify the fitting function $f(\cdot)$ in Eqns. 29 as a power law with a constant coefficient β. This is because the obtained temporary equations consist of derivatives multiplied by variable coefficients, which are power-law functions. Then, the Eqns. 29 can be simplified as:

$$\xi_6 = \beta \mu^{w_1} l^{w_2} V^{w_3} \rho^{w_4} p_0^{w_5}, \tag{31}$$

where $\boldsymbol{w} = [w_1, w_2, w_3, w_4, w_5]^T$ denotes the powers that generate the dimensionless number and are to be determined.

The powers $\boldsymbol{w}$ need to satisfy the following linear system of equations, as explained in Section 1 of the SI:

$$\boldsymbol{D}\boldsymbol{w} = 0. \tag{32}$$

Furthermore, the solutions of the linear system (Eqns. 32) can be written as a linear combination of two basis vectors $\boldsymbol{w_{b1}}$ and $\boldsymbol{w_{b2}}$:

$$\boldsymbol{w} = \gamma_1 \boldsymbol{w_{b1}} + \gamma_2 \boldsymbol{w_{b2}}, \tag{33}$$

where $\boldsymbol{w_{b1}} = [-1, 1, 1, 1, 0]^T$ and $\boldsymbol{w_{b2}} = [-1, 1, -1, 0, 1]^T$.

To summarize, there are three unknown parameters in the identification of ξ_6 and ξ_7, including two basis coefficients (γ_1 and γ_2) and a constant coefficient β. The training data come from the non-zero regression coefficient dataset in Supplementary Table 8. The dataset includes five input parameters (l, V, μ, ρ, and p_0) and one output value for ξ_6 and ξ_7. Applying a pattern search-based optimization method with a grid search range of [-2, 2] and a grid interval of 0.5, the optimized expressions for ξ_6 and ξ_7 can be written as:

$$\xi_6 = \xi_7 = 1.0833 \frac{\mu}{\rho V l} \approx \frac{\mu}{\rho V l}. \tag{34}$$

This form is similar to the reciprocal of the well-known Reynolds number, indicating that the integration of dimensionless learning can directly identify the expression of sparse regression coefficients. By substituting the expressions for ξ_6 and ξ_7 (Eqns. 34) into Eqns. 28, we obtain a consistent dimensionless governing equation for all cases:

$$\frac{\partial \omega}{\partial t} = -u \frac{\partial \omega}{\partial x} - v \frac{\partial \omega}{\partial y} + \frac{1}{\mathrm{Re}} \left(\frac{\partial^2 \omega}{\partial x^2} + \frac{\partial^2 \omega}{\partial y^2} \right), \tag{35}$$

which is identical to the well-known Navier-Stokes equation in a vorticity form.

Furthermore, we test the noisy data effect on this problem. After adding 1% Gaussian noise to the measurements, the identified regression coefficients are (1) case 1, $\xi_{12} = \xi_{19} = -0.9994$, $\xi_6 = \xi_7 = 0.0206$; (2) case 2, $\xi_{12} = \xi_{19} = -0.9909$, $\xi_6 = \xi_7 = 0.0126$; and (3) case 3, $\xi_{12} = \xi_{19} = -0.0.9941$, $\xi_6 = \xi_7 = 0.0111$. Applying dimensionless learning, both ξ_6 and ξ_7 are identified as $1.058 \frac{1}{\mathrm{Re}}$, which is close to the reciprocal of the Reynolds number. Therefore, even with the noisy data effect, the proposed method can identify the governing equation for the system.

7.2 Navier-Stokes equation

In this section, we demonstrate that dimensionless learning in conjunction with symmetric invariant SINDy can be used to identify the Navier-Stokes equation in a Cartesian coordinate system. We performed three series of simulations for the Kármán vortex street problem with three circular cylinders to prepare the original data using Flow3D. The simulation parameters with their values for different cases are shown in Supplementary Table 9. The time-varying measurements include a specific time step (t), two spatial Cartesian coordinates (x and y), and three time-varying measurements (fluid velocity in x direction u, velocity in y direction v, and pressure p).

Supplementary Table 9: **Simulation parameters for the fluid flow through three circular cylinders to identify the Navier-Stokes equation.**

Parameter<br>Unit	l<br>cm	V<br>cm/s	μ<br>kg/cm/s	ρ<br>g/cm^3	p_0<br>Pa	Re<br>1	l_x<br>cm	l_y<br>cm	N_x<br>1	N_y<br>1
Case 1	0.1000	100.00	0.1100	0.0011	8.53×10^5	100	0.9	0.4	500	222
Case 2	0.0950	165.43	0.1040	0.0011	8.53×10^5	170	0.9	0.4	500	222
Case 3	0.1050	170.43	0.1020	0.0011	9.13×10^5	200	0.9	0.4	500	222

Supplementary Table 10: **Identified regression coefficients accompanied by several ground truth dimensionless numbers for the Navier-Stokes problem (clean data).**

Case ID	$\xi_8 = \xi_{13}$	$\xi_6 = \xi_7$	ξ_{17}	Re	1/Re	Eu
Case 1	-0.9759	0.0105	-14127.23	100	0.0100	14545.45
Case 2	-0.9814	0.0063	-3850.28	170	0.0059	3897.63
Case 3	-0.9820	0.0059	-2977.75	200	0.0050	3019.97

Following the same procedures described in Section 7.1, the regression model is defined as:

$$\frac{\partial u^*}{\partial t} = [u^*, uu^*, vu^*, \frac{\partial u^*}{\partial x}, \frac{\partial u^*}{\partial y}, \frac{\partial^2 u^*}{\partial x^2}, \frac{\partial^2 u^*}{\partial y^2}, u\frac{\partial u^*}{\partial x}, u\frac{\partial u^*}{\partial y}, u\frac{\partial^2 u^*}{\partial x^2}, \\ u\frac{\partial^2 u^*}{\partial y^2}, v\frac{\partial u^*}{\partial x}, v\frac{\partial u^*}{\partial y}, v\frac{\partial^2 u^*}{\partial x^2}, v\frac{\partial^2 u^*}{\partial y^2}, p^*, \frac{\partial p^*}{\partial x}]\boldsymbol{\xi}, \tag{36}$$

where $\boldsymbol{\xi}$ is a vector with 17 terms, u^* is the combination of u and v, v^* is the combination of v and u, and p^* is the combination of p and p. It is worth mentioning that u^*, v^*, and p^* are designed for the flipping operation so that we can concatenate the regression libraries for the original and transformed data and train them together. This operation helps to incorporate symmetric invariance into the learning scheme. After the transformation, $u^{*\prime}$ and $v^{*\prime}$ become v^* and u^*, respectively, while $p^{*\prime}$ remains the same as p^*.

Following the same procedure as Section 7.1, we obtain the optimized sparse regression coefficients. There are only five terms with non-zero regression coefficients, as shown in Supplementary Table 10.

Because $\xi_8 = \xi_{13}$ are close to -1, it is reasonable to assign -1 to both ξ_8 and ξ_{13}. Next, we apply dimensionless learning to identify the expression for the other three non-zero coefficients. Using the same pattern search optimization described in Section 7.1, the identified ξ_6 and ξ_7 are $1.0746\frac{\mu}{\rho V l}$, which can be simplified to $\frac{1}{\mathrm{Re}}$. The ξ_{17} is identified as $-0.9729\frac{\mathrm{p_0}}{\rho \mathrm{V}^2}$ and can be simplified as the negative of a well-known dimensionless number, Euler number, Eu. By substituting all non-zero regression coefficients into Eqns. 36, we obtain a consistent governing equation:

$$\frac{\partial u^*}{\partial t} = -u\frac{\partial u^*}{\partial x} - v\frac{\partial u^*}{\partial y} - \mathrm{Eu}\frac{\partial p^*}{\partial x} + \frac{1}{\mathrm{Re}}(\frac{\partial^2 u^*}{\partial x^2} + \frac{\partial^2 u^*}{\partial y^2}), \tag{37}$$

which is identical to the Navier-Stokes momentum equation written for the combination of x and y directions.

We also test the noisy data effect on this problem by imposing 0.5% Gaussian noise to the measurements. The identified non-zero coefficients are shown in Supplementary Table 11.

Using dimensionless learning, we can identify the expression for ξ_6 and ξ_7 as $1.0473\frac{\mu}{\rho V l}$. Similarly, the expression for ξ_{17} is identified as $-0.9687\mathrm{Eu}$. Compared to the clean data results in Supplementary Table 10, ξ_8 and ξ_{13} have higher errors. The ξ_8 and ξ_{13} can be simplified as $-\mathrm{Eu}$, yielding the same equation as Eqns. 37.

It is worth mentioning that we choose a different sampling region for the same data in this problem, but all other procedures remain the same as in the previous example. The identified non-zero regression coefficients for clean and noisy data (1% Gaussian noise) are shown in Supplementary Table 12 and Supplementary Table 13.

Supplementary Table 11: **Identified regression coefficients accompanied by several ground truth dimensionless numbers for the Navier-Stokes example (noisy data).**

Case ID	$\xi_8 = \xi_{13}$	$\xi_6 = \xi_7$	ξ_{17}	Re	1/Re	Eu
Case 1	-0.9668	0.0104	-14139.49	100	0.0100	14545.45
Case 2	-0.9378	0.0063	-3699.04	170	0.0059	3897.63
Case 3	-0.9217	0.0053	-2785.92	200	0.0050	3019.97

Supplementary Table 12: **Identified regression coefficients accompanied by several ground truth dimensionless numbers for the Euler equation example (clean data).**

Case ID	$\xi_8 = \xi_{13}$	ξ_{17}	Re	Eu
Case 1	-1.0436	-14127.23	100	14545.45
Case 2	-1.0016	-3896.14	170	3897.63
Case 3	-1.0177	-3004.48	200	3019.97

From Supplementary Table 12 and Supplementary Table 13, we can see that ξ_8 and ξ_{13} are very close to -1, and ξ_{17} is the only varying regression coefficient. To identify the expression of ξ_{17} for clean and noisy data, we use dimensionless learning and identify the expressions are -0.9671Eu and -0.9661Eu, respectively. By simplifying ξ_{17} to $-\text{Eu}$ and substituting all non-zero regression coefficients into Eqns. 36, we obtain the governing equation as below:

$$\frac{\partial u^*}{\partial t} = -u\frac{\partial u^*}{\partial x} - v\frac{\partial u^*}{\partial y} - \text{Eu}\frac{\partial p^*}{\partial x}, \tag{38}$$

which is identical to the Euler equation. The reason for simplifying the Navier-Stokes equation to the Euler equation is that the viscous effects of the fluid far from the cylinders and walls are negligible in this problem.

7.3 Spring-mass-damper systems

7.3.1 Data generation and preprocessing

The schematic of a spring-mass-damping system is shown in Figure 16.

The system is governed by the equations:

$$\begin{cases} m\frac{d^2x}{dt^2} + kx + c\frac{dx}{dt} = 0, \\ x = \delta, \frac{dx}{dt} = 0 \quad \text{at} \quad t = 0. \end{cases} \tag{39}$$

Supplementary Table 13: **Identified regression coefficients accompanied by several ground truth dimensionless numbers for the Euler equation example (noisy data).**

Case ID	$\xi_8 = \xi_{13}$	ξ_{17}	Re	Eu
Case 1	-1.0361	-14015.25	100	14545.45
Case 2	-0.9946	-3873.86	170	3897.63
Case 3	-1.0088	-2953.95	200	3019.97

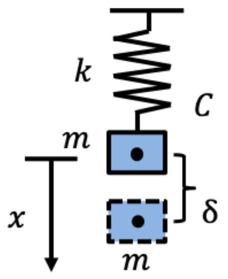

Supplementary Figure 16: **A schematic of the spring-mass-damping system.** A mass m is suspended from a spring with an initial displacement δ, a spring constant k, and a damping coefficient c.

Supplementary Table 14: **Key parameters and their values for different cases to generate data for the spring-mass-damping system problem.**

Parameter<br>Unit	k<br>N/m	m<br>kg	δ<br>m	c<br>Ns/m
Case 1	1.0	1.0	0.1	0.100
Case 2	0.5	0.8	0.05	0.050
Case 3	0.1	0.1	0.02	0.004
Case 4	0.2	0.2	0.07	0.020

The analytical solution for Equ. 39 with a small damping coefficient c is:

$$x(t) = Ae^{-\frac{c}{2m}t}\sin(\sqrt{\frac{k}{m} - (\frac{c}{2m})^2}t + \varphi), \tag{40}$$

where $A = \sqrt{\delta^2 + (\frac{\frac{dx}{dt}|_{t=0} + \frac{c}{2m}\delta}{\sqrt{\frac{k}{m} - (\frac{c}{2m})^2}})^2}$, and $\varphi = \arctan\frac{\sqrt{\frac{k}{m} - (\frac{c}{2m})^2}\delta}{\frac{dx}{dt}|_{t=0} + \frac{c}{2m}\delta}$.

Based on the analytical solution (Equ. 40), we generate three sets of data with different k, m, δ, and c, which are shown in Supplementary Figure 17. For each set of data, the starting and ending times are set to 0 s and 20 s, respectively. The number of sampled data points is 800. The detailed information can be found in Supplementary Table 14.

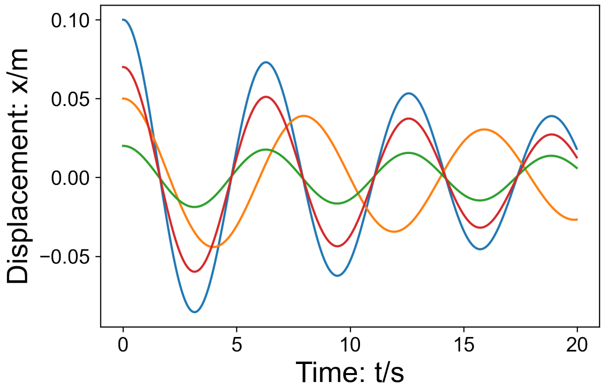

Supplementary Figure 17: **Plot showing displacements for four sets of generated data for the spring-mass-damping systems.**

Supplementary Table 15: **Identified regression coefficients for the spring-mass-damping systems (clean data).**

Case ID	ξ_1	ξ_2
Case 1	-10.0086	-10.0170
Case 2	-10.0054	-16.0171
Case 3	-25.0214	-25.0428
Case 4	-10.0086	-10.0170

7.3.2 Temporary governing equation discovery

For each set of data, we apply SINDy to find the temporary governing equation. The regression model is defined as:

$$\frac{dx}{dt} = [x, \frac{d^2x}{dt^2}, x^2, x\frac{dx}{dt}, x\frac{d^2x}{dt^2}]\boldsymbol{\xi}, \tag{41}$$

where $\boldsymbol{\xi}$ is a vector with five terms.

The optimized sparse regression coefficients have only two non-zero terms: x and $\frac{d^2x}{dt^2}$. The non-zero regression coefficients are shown in Supplementary Table 15. The dimensions of ξ_1 and ξ_2 are $[\mathrm{T}]$ and $[\mathrm{T}^{-1}]$, respectively, in order to make Eqns. 41 dimensionally homogeneous. It is worth mentioning that the coefficients in Supplementary Table 15 are different from case to case even though all the data from the same dynamic system with different parameters. This means that if we only use SINDy, we end up with multiple different governing equations for the same dynamic system.

7.3.3 Dimensionless learning

Dimensionless learning is introduced to address the aforementioned issue: inconsistent governing equations for the same dynamic system. It is designed to find the explicit expression for each varying regression coefficient from SINDy. Then, multiple governing equations can be summarized into one consistent parameterized governing equation. The detailed procedures are described below.

First, we explore the parameter space for ξ_1. The causal relationship for ξ_1 can be expressed as:

$$\xi_1 = f(m, k, \delta, c), \tag{42}$$

where the fitting function $f(\cdot)$ is assumed to be a power law with a constant coefficient β. The power law index is defined as a vector $\boldsymbol{w} = [w_1, w_2, w_3, w_4]$.

The dimension matrix $\boldsymbol{D}$ for Eqns. 42 is:

$$\begin{bmatrix} 1 & 1 & 0 & 1 \\ 0 & 0 & 1 & 0 \\ 0 & -2 & 0 & -1 \end{bmatrix} \tag{43}$$

The dimensions of m, k, δ, and c are represented by the first to last column. From the first row to the last row, it represents the dimensions of mass, length, and time.

The basis vectors for $\boldsymbol{w}$ can be written as:

$$\boldsymbol{w} = \gamma \boldsymbol{w_{b1}} + \boldsymbol{w_{b2}}, \tag{44}$$

where $\boldsymbol{w_{b1}} = [1, 1, 0, -2]^T$ and $\boldsymbol{w_{b2}} = [1, 0, 0, -1]^T$.

After using the pattern search-based optimization method, the identified expression for ξ_1 is $-1.000\frac{k}{c}$. Similarly, the expression for ξ_2 is identified as $-1.001\frac{m}{c}$. By substituting these two regression coefficients into Eqns. 41, we obtain a consistent governing equation for all cases:

$$\frac{dx}{dt} = -\frac{k}{c}x - \frac{m}{c}\frac{d^2x}{dt^2}, \tag{45}$$

which is identical to Eqns 39 after rearranging the terms into one side.

Supplementary Table 16: **Identified regression coefficients for the spring-mass-damping systems (noisy data).**

Case ID	ξ_1	ξ_2
Case 1	-10.6596	-10.8786
Case 2	-9.1255	-14.6939
Case 3	-24.8092	-25.3340
Case 4	-10.6597	-10.8787

Furthermore, we test the noisy data effects in this problem by imposing 4% Gaussian noise. We follow two steps to reduce the noisy data effect. In the first step, we fit a third-order polynomial to local measurements (60 points) and use the fitted function to reconstruct the original measurements. Next, the Total Variation Regularized Numerical Differentiation (TVDiff) algorithm [17] is used to calculate the first and second derivatives.

The identified non-zero regression coefficients for noisy data are shown in Supplementary Table 16. Following the same procedure as with the clean data, the expressions for ξ_1 and ξ_2 are $-0.9997\frac{k}{c}$ and $-1.005\frac{m}{c}$, respectively. These two identified expressions are very close to the ground truth $-\frac{k}{c}$ and $-\frac{m}{c}$. Therefore, even with the noisy data effect, the proposed method can identify the governing equation for spring-mass-damper systems.

7.4 Dynamic response of impulsively loaded plastic beams

7.4.1 Data generation and preprocessing

The structural impact problem is critical for structures subjected to dynamic loads since large plastic deformation can lead to structural failures [18]. As a special case, the dynamic response of a rigid-plastic beam subjected to a uniform velocity impulse can be solved analytically. Supplementary Figure 18 shows the schematic of a simply supported, rigid-plastic beam with unit width under an initial impulsive loading V_0. The physical properties of the beam include material density ρ, beam height H, beam length $2L$, and yield strength σ_0.

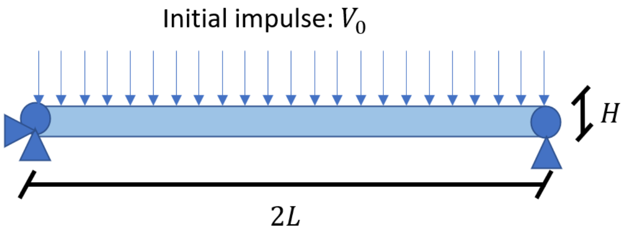

Supplementary Figure 18: **Impulsively loaded simply supported beam.**

The dynamic response of this impulsively loaded rigid-plastic beam can be expressed using the following PDE [19]:

$$\frac{\partial^2 M}{\partial x^2} = \rho H \frac{\partial V}{\partial t}, \tag{46}$$

where M is the moment acting on the beam, V is the velocity of beam deflection. The analytical solution of Eqn. 46 contains two separate stages and is discussed in detail in [18, 20]. As shown in Supplementary Table 17, four sets of data using different values for the key parameters including ρ, H, L, V_0, and σ_0 have been generated to discover the governing PDE. As an example, Supplementary Figure 19 shows the solution for Case 2. Before discovering the differential equation, M, v, x, and t are non-dimensionalized with respect to the corresponding characteristic values: $M^* = \frac{4M}{\sigma_0 H^2}$ is the dimensionless moment; $v^* = \frac{v}{V_0}$ is the dimensionless beam velocity; $x^* = \frac{x}{L}$ is the dimensionless position; $t^* = \frac{tV_0}{H}$ is the dimensionless time.

Supplementary Table 17: **Data generation for the impulsively loaded plastic beam.**

Parameter<br>Unit	ρ<br>$\mathrm{t/mm^3}$	H<br>mm	L<br>mm	V_0<br>$\mathrm{mm/s^2}$	σ_0<br>MPa
Case 1	2×10^{-9}	100	2.5×10^4	1000	100
Case 2	7×10^{-9}	50	3.5×10^4	700	700
Case 3	3×10^{-9}	30	3.0×10^4	800	500
Case 4	5×10^{-9}	20	2.0×10^4	500	400

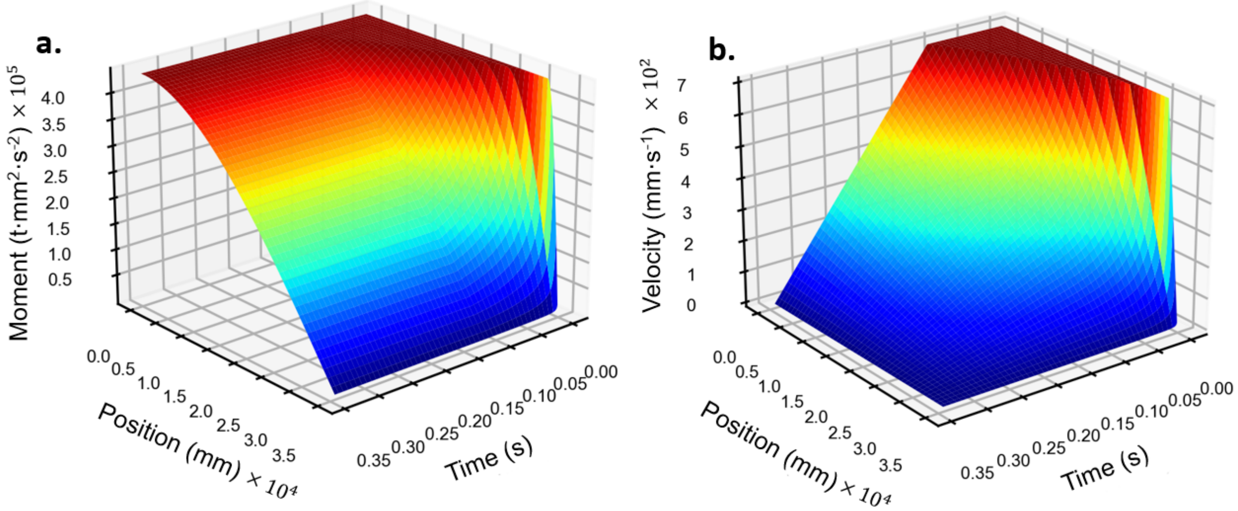

Supplementary Figure 19: **Data generated for an impulsively loaded plastic beam (Case 2). (a)**: moment measurements M. **(b)**: velocity measurements v.

7.4.2 Temporary governing equation discovery

Following the same procedures described in Section 7.1, the regression library can be created as follows:

$$\frac{\partial^2 M^*}{\partial x^{*2}} = [v^*, M^*, \frac{\partial v^*}{\partial t^*}, \frac{\partial M^*}{\partial t^*}, \frac{\partial v^*}{\partial x^*}, \frac{\partial M^*}{\partial x^*}, \frac{\partial^2 v^*}{\partial x^{*2}}]\boldsymbol{\xi}, \tag{47}$$

where $\boldsymbol{\xi}$ is the coefficient vector with seven terms. After sparse optimization, only one coefficient is non-zero and is shown in Supplementary Table 18.

7.4.3 Dimensionless learning

Because the identified coefficient ξ_3 varies with the parameter settings, the proposed dimensionless learning is used to identify the analytical expression of ξ_3. The relationship between ξ_3 and other parameters can be expressed as:

$$\xi_3 = f(\rho, H, L, V_0, \sigma_0), \tag{48}$$

where the fitting function $f(\cdot)$ is assumed to be a power law with a constant coefficient, as in the previous sections.

In this problem, the corresponding dimension matrix is:

$$\begin{bmatrix} 1 & 0 & 0 & 0 & 1 \\ -3 & 1 & 1 & 1 & -1 \\ 0 & 0 & 0 & -1 & -2 \end{bmatrix} \tag{49}$$

Supplementary Table 18: **Identified non-zero regression coefficients for impulsively loaded beams (clean data).**

Case ID	ξ_3
Case 1	5.0153
Case 2	9.6331
Case 3	15.4277
Case 4	12.5380

Supplementary Table 19: **Identified non-zero regression coefficients for impulsively loaded beams (noisy data).**

Case ID	ξ_3
Case 1	4.9129
Case 2	9.4315
Case 3	15.0665
Case 4	12.2690

where the dimensions of $\rho, H, L, V_0, \sigma_0$ are listed from the first to last column. The first, second and third rows represent the dimensions of mass, length, and time, respectively.

The power law index vector $\boldsymbol{w}$ can be expressed as:

$$\boldsymbol{w} = \gamma_1 \boldsymbol{w_{b1}} + \gamma_2 \boldsymbol{w_{b2}}, \tag{50}$$

where $\boldsymbol{w_{b1}} = [0, -1, 1, 0, 0]^T$ and $\boldsymbol{w_{b2}} = [1, 0, 0, 2, -1]^T$.

Using grid search-based optimization method, the identified ξ_3 can be expressed as:

$$\xi_3 = 4.0147 \frac{\rho V_0^2}{\sigma_0} (\frac{L}{H})^2, \tag{51}$$

which is almost identical to the well-known dimensionless number called response number $\mathrm{Rn} = \frac{4\rho V_0^2}{\sigma_0}(\frac{L}{H})^2$ [20].

By substituting the Rn into Eqns. 47, we obtain the final parameterized differential equation:

$$\frac{\partial^2 M^*}{\partial x^{*2}} = \mathrm{Rn} \frac{\partial v^*}{\partial t^*}, \tag{52}$$

which is a dimensionless form of Eqns. 46 with the response number Rn, indicating the effectiveness of the integration of dimensionless learning with SINDy.

Furthermore, in order to test the performance of the proposed method for data contaminated by noise, 2% Gaussian noise is superimposed to the original clean data. With the same procedure discussed in section 6.4.2, the identified regression coefficients and response number can be obtained, as listed in Supplementary Table 19. Similarly, based on the identified regression coefficients, we can obtain the expression of $\xi_3 = 3.9255 \frac{\rho V_0^2}{\sigma_0}(\frac{L}{H})^2$, which is also close to Rn. Consequently, the proposed scheme can successfully discover the dimensionless governing equation that captures the dynamic response of the rigid-plastic beam under impulsive loading.

7.5 Regression library suggestions

Building a regression library with all the required candidates relies on the researchers' experience and understanding of the problem. If some terms are known to be important to the problems, more specific terms can be included, limiting the

regression library to a smaller one. For example, convective and diffusion terms are frequently used in the governing equations of fluid mechanics. It is reasonable to include these terms in the library.

In addition, if there is some prior knowledge about the relationship between different terms, the number of learnable coefficients can be reduced. For example, for a 2-dimensional fluid mechanics problem, we can assume that the two convective and diffusion terms have the same coefficients. It is worth noting that the proposed symmetric invariance does not explicitly include this physical constraint but achieves it implicitly.

In a more general and unknown problem, we suggest including linear terms first, such as the original measurements and their first and second order derivatives. If the fitting performance is ideal, we can stop involving more terms and obtain the best differential equations. Otherwise, we will keep adding quadratic and other high-order terms and testing the fitting performance. The library will be updated until the best performance model is discovered.

Supplementary References

[1] Edgar Buckingham. On physically similar systems; illustrations of the use of dimensional equations. *Physical review*, 4(4):345, 1914.

[2] Ernst Gőbel, Ian Mills, and Andrew Wallard. The international system of units (si). 2006.

[3] David Donoho, Hossein Kakavand, and James Mammen. The simplest solution to an underdetermined system of linear equations. In *2006 IEEE International Symposium on Information Theory*, pages 1924–1928. IEEE, 2006.

[4] R Fletcher. Practical methods of optimization, 1987.

[5] Tianqi Chen and Carlos Guestrin. Xgboost: A scalable tree boosting system. In *Proceedings of the 22nd acm sigkdd international conference on knowledge discovery and data mining*, pages 785–794, 2016.

[6] Takuya Iwanaga, William Usher, and Jonathan Herman. Toward SALib 2.0: Advancing the accessibility and interpretability of global sensitivity analyses. *Socio-Environmental Systems Modelling*, 4:18155, May 2022.

[7] Jon Herman and Will Usher. SALib: An open-source python library for sensitivity analysis. *The Journal of Open Source Software*, 2(9), jan 2017.

[8] Il'ya Meerovich Sobol'. On the distribution of points in a cube and the approximate evaluation of integrals. *Zhurnal Vychislitel'noi Matematiki i Matematicheskoi Fiziki*, 7(4):784–802, 1967.

[9] Daniel Svozil, Vladimir Kvasnicka, and Jiri Pospichal. Introduction to multi-layer feed-forward neural networks. *Chemometrics and intelligent laboratory systems*, 39(1):43–62, 1997.

[10] Keinosuke Fukunaga and Patrenahalli M. Narendra. A branch and bound algorithm for computing k-nearest neighbors. *IEEE transactions on computers*, 100(7):750–753, 1975.

[11] Leo Breiman. Random Forests. *Machine Learning*, 45(1):5–32, October 2001.

[12] Osborne Reynolds. Xxix. an experimental investigation of the circumstances which determine whether the motion of water shall be direct or sinuous, and of the law of resistance in parallel channels. *Philosophical Transactions of the Royal society of London*, (174):935–982, 1883.

[13] Inc. Flow Science. *FLOW-3D, Version 12.0*. Santa Fe, NM, 2019.

[14] Uday Shankar Shanthamallu, Andreas Spanias, Cihan Tepedelenlioglu, and Mike Stanley. A brief survey of machine learning methods and their sensor and iot applications. In *2017 8th International Conference on Information, Intelligence, Systems & Applications (IISA)*, pages 1–8. IEEE, 2017.

[15] Samuel H Rudy, Steven L Brunton, Joshua L Proctor, and J Nathan Kutz. Data-driven discovery of partial differential equations. *Science advances*, 3(4):e1602614, 2017.

[16] Ian Knowles and Robert J Renka. Methods for numerical differentiation of noisy data. *Electron. J. Differ. Equ*, 21:235–246, 2014.

[17] Rick Chartrand. Numerical differentiation of noisy, nonsmooth data. *International Scholarly Research Notices*, 2011, 2011.

[18] Norman Jones. *Structural impact*. Cambridge university press, 2011.

[19] Norman Jones. Influence of strain-hardening and strain-rate sensitivity on the permanent deformation of impulsively loaded rigid-plastic beams. *International Journal of Mechanical Sciences*, 9(12):777–796, 1967.

[20] Ya-Pu Zhao. Suggestion of a new dimensionless number for dynamic plastic response of beams and plates. *Archive of Applied Mechanics*, 68(7):524–538, 1998.
